# RNA localization in confined cells depends on cellular mechanical activity and contributes to confined migration

**DOI:** 10.1016/j.isci.2022.103845

**Published:** 2022-02-01

**Authors:** Rebecca A. Moriarty, Stavroula Mili, Kimberly M. Stroka

**Affiliations:** 1 Laboratory of Cellular and Molecular Biology, Center for Cancer Research, National Cancer Institute, NIH, Bethesda, MD 20892, USA; 2 Fischell Department of Bioengineering, University of Maryland College Park, College Park, MD 20742, USA; 3 Maryland Biophysics Program, University of Maryland College Park, College Park, MD 20742, USA; 4 Center for Stem Cell Biology & Regenerative Medicine, University of Maryland Baltimore, Baltimore, MD 21202, USA; 5 Marlene and Stewart Greenebaum Comprehensive Cancer Center, University of Maryland Baltimore, Baltimore, MD 21202, USA

**Keywords:** Biological sciences, Bioengineering, Cell biology, Biophysics

## Abstract

Cancer cells experience mechanical confining forces during metastasis and, consequently, can alter their migratory mechanisms. Localization of numerous mRNAs to cell protrusions contributes to cell polarization and migration and is controlled by proteins that can bind RNA and/or cytoskeletal elements, such as the adenomatous polyposis coli (APC). Here, we demonstrate that peripheral localization of APC-dependent RNAs in cells within confined microchannels is cell type dependent. This varying phenotype is determined by the levels of a detyrosinated tubulin network. We show that this network is regulated by mechanoactivity and that cells with mechanosensitive ion channels and increased myosin II activity direct peripheral localization of the *RAB13* APC-dependent RNA. Through specific mislocalization of the *RAB13* RNA, we show that peripheral RNA localization contributes to confined cell migration. Our results indicate that a cell’s mechanical activity determines its ability to peripherally target RNAs and utilize them for movement in confinement.

## Highlights

• Peripheral localization of APC-dependent RNAs in confinement depends on cell type
• RNA localization in confined cells is controlled by the mechanoactivity of cells
• RNA localization phenotype is influenced by the detyrosinated tubulin network
• Peripheral RNA accumulation functionally contributes to confined cell migration

## Graphical abstract

**Figure:**
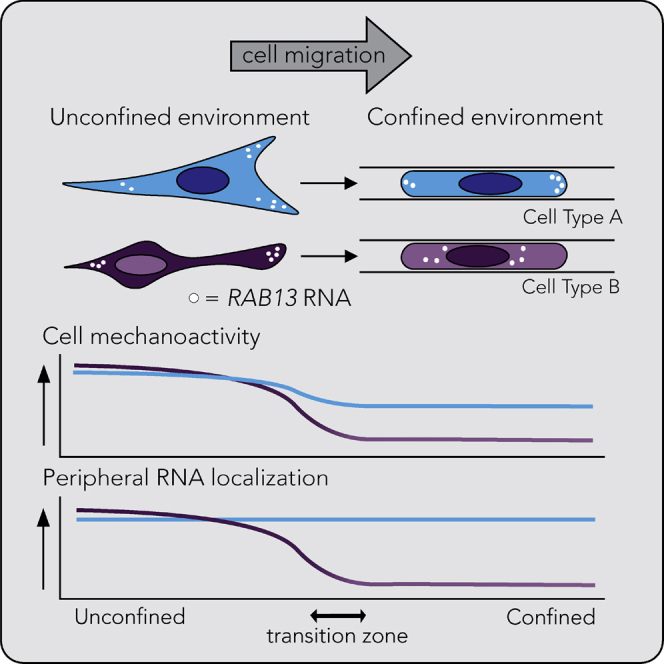

## Introduction

Cell behavior can be influenced by biochemical as well as mechanical cues (Gasiorowski et al., 2013; Spill et al., 2016). Among the latter, cancer cells experience mechanical confinement *in vivo* in a multitude of environments during metastasis. For example, as cancer cells migrate out from the primary tumor, they are confined by the surrounding extracellular matrix, composed of collagen fibers, where pore spacing can range from 1 to 30 μm in diameter (Alexander et al., 2008; Friedl and Alexander, 2011). Generally, pores between 7 and 70 μm^2^ in cross-sectional area are considered confining because they require nuclear deformation for cells to fit into (Wolf et al., 2013). In addition, tumor cells experience confinement as they intravasate into, circulate through, and extravasate from the bloodstream, where they can travel through narrow capillaries as small as 3–4 μm in diameter (Alexander et al., 2013; Weigelin et al., 2012). Mounting evidence over the past decade has revealed that confinement impacts cell migration mechanisms and phenotypes (Paul et al., 2016, 2017). For example, traditional haptokinetic actomyosin contractility is often utilized by migrating cells on unconfined 2D substrates, whereas in confinement, cells can employ ameboid-based methods relying on myosin II-mediated contractility (Hung et al., 2013; Liu et al., 2015). Other migratory mechanisms in confinement have included (but are certainly not limited to) a nuclear-piston propeller method (Petrie et al., 2014, 2017); an osmotic engine mode, involving aquaporin-mediated water flux (Stroka et al., 2014); or a dependence on microtubule dynamics (Balzer et al., 2012).

Many RNAs are enriched in protrusive regions of migrating cells (Herbert and Costa, 2019). This spatial accumulation can be controlled through targeting sequences found within the mRNA transcripts (Andreassi and Riccio, 2009; Jansen, 2001). These localization elements are thought to direct association with molecular motors such as kinesins or myosins, usually through recognition by specific RNA binding proteins, in order to direct movement along cytoskeletal elements within the cell (Bullock, 2011; Jansen, 2001; Medioni et al., 2012). RNAs can be targeted to protrusions in different ways. In one pathway, RNAs are directed to peripheral protrusions in association with the kinesin motor KIF1C (Pichon et al., 2020). This pathway transports RNAs such as *RAB13* and the *KIF1C* RNA itself. Peripheral localization of these RNAs additionally requires the tumor suppressor protein adenomatous polyposis coli (APC) and stable detyrosinated microtubules (Mili et al., 2008; Wang et al., 2017; Yasuda et al., 2017). A different pathway, which relies on the RNA-binding protein LARP6, directs RNAs encoding ribosomal proteins to protrusions (Dermit et al., 2020). Finally, transport of the β-actin mRNA to protrusions relies on ZBP1 and involves both microtubules and actin filaments as well as different myosins and kinesins, depending on the cell type (Song et al., 2015; Nalavadi et al., 2012; Condeelis and Singer, 2005).

Protrusion-localized RNAs are functionally important for cell migration. Indeed, inhibiting translation at protrusions leads to protrusion destabilization (Mardakheh et al., 2015). Furthermore, preventing protrusion localization of specific RNAs impedes the efficiency of cell migration in various 2D *in vitro* models (Moissoglu et al., 2020; Shestakova et al., 2002; Wang et al., 2017). Localized RNAs of the APC-dependent group are additionally important for collective 3D invasion of multicellular cancer spheroids, as well as for directing blood vessel morphogenesis *in vivo* (Chrisafis et al., 2020; Costa et al., 2020).

RNA localization at protrusions can be affected by the mechanical properties of the extracellular matrix (ECM) and actomyosin contractility. Mechanical tension at sites of integrin attachment promotes localization of polyadenylated RNAs (Chicurel et al., 1998). Accumulation of APC-dependent RNAs at the front of collectively invading cell strands requires integrin-mediated contact with the ECM and coincides with areas of high laminin concentration (Chrisafis et al., 2020). Furthermore, stiff substrates promote peripheral localization of APC-dependent RNAs (Wang et al., 2017). Stiff surfaces promote actomyosin contractility, formation of fibrillar actin, and focal adhesions (Discher et al., 2005; Eyckmans et al., 2011; Geiger et al., 2009; Schwartz, 2010). Actomyosin contractility promotes the formation of detyrosinated microtubules, and consequently APC-dependent RNA localization. The underlying signaling pathway involves the GTPase RhoA and the formin mDia1, factors central in organizing actin and microtubule outgrowth at cell protrusions (Bartolini and Gundersen, 2010; Bartolini et al., 2016; Wang et al., 2017; Zaoui et al., 2008). Contractility-dependent localization does not likely apply to all protrusion-localized RNAs. Indeed, ribosomal protein mRNAs become preferentially enriched in protrusions that are apparently less contractile, characterized by low levels of active myosin (Wang et al., 2017).

In this study, we sought to understand how RNA localization is controlled by the mechanical cue of confinement. We employed microfluidic microchannel devices to model unconfined and confined environments. We imaged RNAs in two cells lines: (1) the MDA-MB-231 metastatic breast cancer cell line, which can use microtubule-based methods (Balzer et al., 2012) and/or an osmotic engine model (Stroka et al., 2014) to migrate in confined spaces, and (2) the A375 metastatic melanoma line, which utilizes ameboid-based myosin II contractility in confinement (Hung et al., 2013). We show that RNA localization patterns change in confinement in a cell type-dependent manner. We focused on the APC-dependent *RAB13* RNA, which remains peripherally localized only in confined A375 cells. We show that *RAB13* RNA localization depends on detyrosinated microtubules and the mechanical state of cells. Specifically, the Piezo1 mechanosensitive ion channel and myosin II activity act redundantly to upregulate the detyrosinated tubulin network in confinement, leading to peripheral *RAB13* RNA localization. This mechanism functionally contributes to confined migration of A375 cells. By contrast, confined MDA-MB-231 cells do not exhibit peripheral *RAB13* RNA and do not rely on it for migration in narrow spaces. This work highlights the contribution of peripherally localized RNAs during confined migration and indicates that different migration modes adopted by cancer cells to navigate through confined spaces variably depend on specific localized mRNAs.

## Results

### RNA localization patterns are altered in cells in confined microchannels

**Figure 1:**
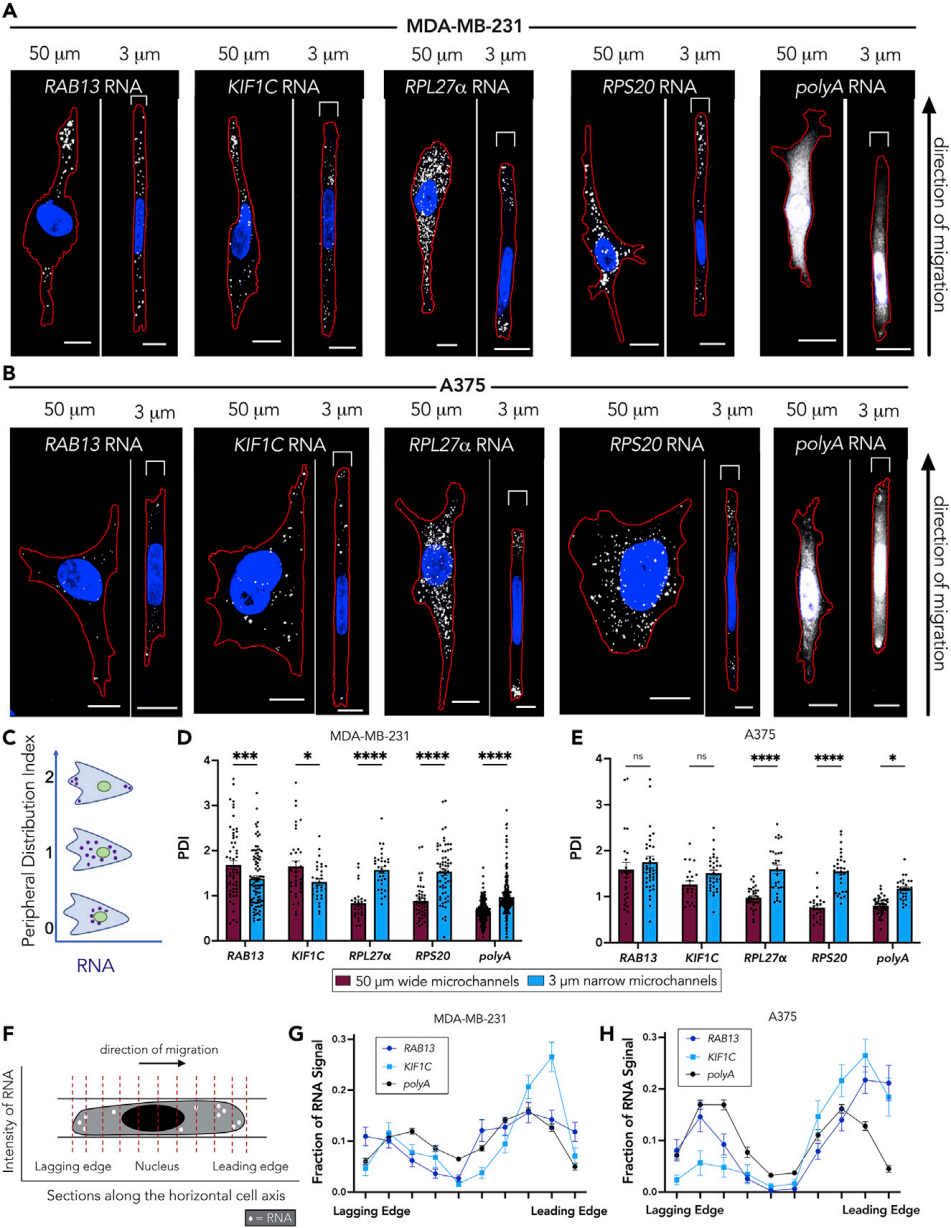
RNA localization patterns are altered in a cell type-dependent manner in confinement (A and B) Representative maximum intensity z-projected FISH images of *RAB13*, *KIF1C*, *RPL27*α, *RPS20*, and *polyA* RNAs in (A) MDA-MB-231 and (B) A375 cells in either 50-μm wide or 3-μm narrow microchannels. RNA signal is shown in white, nucleus in blue, and cell outline in red. Brackets above cells in 3 μm groups denote the microchannel width. The leading edge of the cells (determined by the side of chemoattractant addition) is toward the top of the page. (C) Schematic showing PDI index values of the indicated hypothetical RNA distributions. A more peripherally enriched RNA exhibits a PDI > 1, a diffuse RNA exhibits a PDI = 1, and a perinuclear RNA a PDI < 1. (D and E) PDI calculations of *RAB13*, *KIF1C*, *RPL27*α, *RPS20*, and *polyA* RNAs for (D) MDA-MB-231 cells or (E) A375 cells. Dots on graphs in (D and E) represent individual cells pooled from at least three independent experiments (n > 25). Note that the PDI of polyA RNA, which is used as a proxy for the cytosolic volume, is slightly higher in cells in the 3-μm narrow microchannels. This is likely because in confined cells the cytosolic volume is more equally distributed throughout the cell body, whereas in unconfined cells most of the cytosol is found perinuclearly, leading to higher and lower PDIs, respectively. This difference is not sufficient to account for the more substantial PDI increase observed for r-protein mRNAs in the 3-μm narrow microchannels. (F) Schematic showing segmentation of the cell in the direction of migration, from the lagging edge through the nucleus to the leading edge. The intensity of the RNA signal was measured within ten evenly spaced sections starting and ending at the cell boundaries. (G and H) Quantification of the intensity of APC-dependent RNAs (*RAB13, KIF1C*) compared with *polyA* RNA in (G) MDA-MB-231 and (H) A375 cells along the length of cells in 3-μm narrow microchannels (n > 25). Bars represent mean ± SE. Scale bars: 10 μm. p Values represent ∗<0.05, ∗∗∗<0.001, ∗∗∗∗<0.0001; two-way ANOVA with Šídák’s test.

To examine the effect of confinement on mRNA localization, we seeded cells into microchannels of two distinct widths, either 50 μm in width by 10 μm in height to represent a relatively wide, unconfined environment, or 3 μm in width by 10 μm in height to represent a narrow, confined environment. The latter requires cells to remodel their nucleus to enter, as the cross-sectional area of this environment is below the “nuclear limit” (Denais et al., 2016; Wolf et al., 2013). We chose to compare two cell lines, MDA-MB-231 and A375, as they seem to use two separate mechanisms to migrate in confined microchannels (Balzer et al., 2012; Hung et al., 2013; Stroka et al., 2014). We used fluorescence *in situ* hybridization to assess the distribution of four different mRNAs, *RAB13* and *KIF1C* (APC dependent) and *RPL27*α and *RPS20* (APC independent) in both cell lines (Figures 1A and 1B). To quantify RNA patterns, we measured a peripheral distribution index (PDI), which describes the normalized distance of an RNA population from the centroid of the nucleus. The values are also internally normalized to account for differences in cell size and shape (Figure 1C; Stueland et al., 2019). The MDA-MB-231 cells displayed a peripheral RNA enrichment of the two APC-dependent mRNAs in the 50-μm wide microchannels (Figure 1D), consistent with previously published data on 2D surfaces (Mardakheh et al., 2015; Moissoglu et al., 2020). For cells in the 3-μm narrow microchannels, the APC-dependent mRNAs had a more perinuclear distribution, resulting in a significantly lower PDI. In contrast to the APC-dependent mRNAs, the APC-independent mRNAs (*RPL*27α and *RPS20*) were less peripheral in unconfined environments (Figures 1A and 1D [Wang et al., 2017]). However, for cells in the 3-μm narrow microchannels, the APC-independent RNAs were significantly more peripherally enriched. Therefore, distinct mRNA species undergo opposing changes in their distributions as MDA-MB-231 cells enter confinement.

The A375 cells also displayed a peripheral APC-dependent RNA enrichment in the 50-μm wide microchannels (Figure 1E), consistent with the broadly observed localization of these RNAs in various cell types (Mardakheh et al., 2015; Mili et al., 2008; Moissoglu et al., 2019, 2020; Wang et al., 2017). Interesting, in contrast to what we observed in MDA-MB-231 cells, A375 cells maintained the same degree of peripheral APC-dependent RNA enrichment in confinement. The APC-independent RNAs exhibited a similar behavior in both cell types with a significantly increased peripheral distribution in the 3-μm narrow microchannels. We believe that this increased peripheral distribution is the result of specific RNA targeting and cannot be accounted for by non-specific, overall redistribution of the cytoplasmic volume, which we assessed through detection of total polyadenylated (*polyA*) RNA in the same cells (Figures 1A–1E). Overall, r-protein mRNAs (APC-independent) consistently became more peripheral in confinement, whereas localization of APC-dependent mRNAs is maintained or not in confinement depending on the cell type.

To assess whether the observed peripheral RNA localization is uniform around the cell or whether it exhibits polarity, we measured an RNA polarization index for each cell, which is defined as the normalized distance between the centroid of the overall RNA signal and the centroid of the cell body (Park et al., 2012; Stueland et al., 2019). In MDA-MB-231 cells, APC-dependent RNAs (*RAB13* and *KIF1C*) exhibited a similar polarization index between 50-μm wide and 3-μm narrow microchannels, whereas, in contrast, the polarization of these RNAs was significantly increased in confined A375 cells. The APC-independent r-protein mRNAs exhibited an increased polarization upon confinement in both cell types (Figures S1A and S1B). Nevertheless, this type of polarization metric does not allow us to distinguish the particular site of RNA accumulation and its potential correlation with the direction of migration.

Cells in the 3-μm narrow microchannels are linearly confined and their direction of migration is specified by the site of chemoattractant addition, as indicated by the direction of the migration arrow in Figures 1A and 1B. Thus, we can segment the cells perpendicular to their direction of migration and measure RNA presence along the lagging to leading edge axis (Figure 1F). Of interest, although r-protein mRNAs showed an increased polarization index in both cell types (Figures S1A and S1B), they did not exhibit a consistent preferential distribution toward either the leading or lagging edges (Figures S1C and S1D). It therefore appears that r-protein mRNAs polarize either toward the front or the back of individual cells and when averaged across a population of cells the resulting overall distribution lacks a preference for either side. Although the implications of this behavior, in the context of ribosome function or translation, might be interesting to explore, we think this observation indicates that peripheral localization of r-protein mRNAs is likely not directly linked to the directionality of cell movement in confined spaces.

On the other hand, the APC-dependent RNAs revealed an interesting directional behavior. In MDA-MB-231 cells, which did not maintain peripheral localization of *RAB13* or *KIF1C* RNAs in confinement (Figure 1D), these RNAs also did not preferentially polarize toward the leading or lagging edges (Figure 1G). In contrast, in A375 cells, where *RAB13* and *KIF1C* RNAs were peripheral in cells in confinement (Figure 1E), they also preferentially accumulated toward the leading edge (Figure 1H). Given that localization of APC-dependent RNAs is important in unconfined migration (Moissoglu et al., 2020; Wang et al., 2017), we reasoned that this result might indicate that APC-dependent RNAs have a differential regulation and contribution in confined migration depending on the cell type. Given the known involvement of the *RAB13* mRNA in unconfined cell migration, we focused on it and sought to understand the mechanism leading to its differential distribution in confined MDA-MB-231 or A375 cells and to assess its functional role in confined migration of these cell types.

### Detyrosinated tubulin network drives *RAB13* RNA peripheral localization in confinement

**Figure 2:**
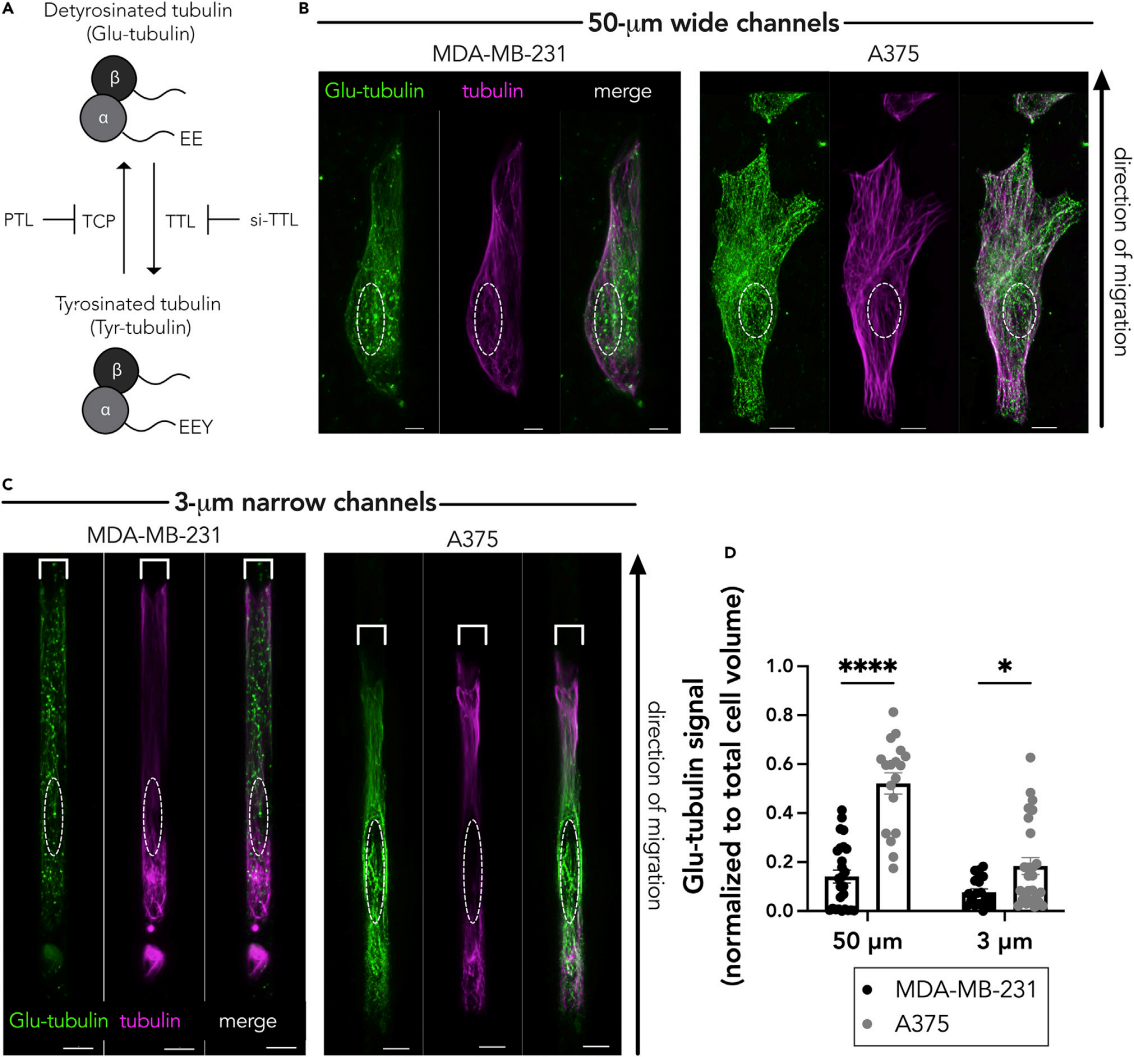
MDA-MB-231 cells have less detyrosinated (Glu) tubulin than A375 cells (A) Schematic representing the tubulin tyrosination and detyrosination cycle and the enzymes involved. The C-terminal tyrosine of α-tubulin can be removed by a tubulin carboxypeptidase (TCP), an enzyme that can be inhibited by parthenolide (PTL). The tyrosine can be added back through the action of tubulin tyrosine ligase (TTL), whose expression can be knocked down through targeted siRNA (si-TTL). (B and C) Representative immunofluorescence images of Glu-tubulin and tubulin in MDA-MB-231 or A375 cells in (B) 50-μm wide microchannels or (C) 3-μm narrow microchannels. Images are snapshots of 3D view renderings. White dashed lines indicate positions of nuclei. Scale bars: 5 μm. (D) Quantification of Glu-tubulin signal (diffuse background signal was thresholded out and normalized to total cell volume) in the indicated cells. Dots represent individual cells pooled from three independent experiments (n = 20–26); bars represent mean ± SE. p Values: ∗<0.05, ∗∗∗∗<0.0001; two-way ANOVA with Šídák's test.

Prior studies have shown that *RAB13* and other APC-dependent RNAs rely on detyrosinated (Glu)-microtubules for their peripheral localization (Mili et al., 2008; Wang et al., 2017; Yasuda et al., 2017). Detyrosinated (Glu)-microtubules are a subset of stable microtubules that arise upon removal of the C-terminal tyrosine from the tail of the α-tubulin subunit (Figure 2A). We therefore hypothesized that differences in APC-dependent RNA distribution between confined MDA-MB-231 and A375 cells could be driven by differences in the Glu-tubulin network. In the 50-μm wide microchannels, there was a detectable detyrosinated tubulin and α-tubulin network in both the MDA-MB-231 and A375 cells (Figure 2B; Video S1), consistent with the peripheral RNA localization in both cases. However, in the 3-μm narrow microchannels, the Glu-tubulin network appeared reduced and fragmented in the MDA-MB-231 cells, in direct contrast to the A375 cells, which maintained a higher Glu-tubulin staining (Figure 2C; Video S2). The total α-tubulin network in the 3-μm-wide microchannels appeared similar in both cell lines. Quantification of the detyrosinated tubulin signal indeed showed that MDA-MB-231 cells exhibit a lower Glu-tubulin signal than the A375 cells (Figure 2D).

Video S1. Representative immunofluorescence image stacks of Glu-tubulin and tubulin in MDA-MB-231 or A375 cells in 50-μm wide microchannels, related to Figure 2B

Video S2. Representative immunofluorescence image stacks of Glu-tubulin and tubulin in MDA-MB-231 or A375 cells in 3-μm narrow microchannels, related to Figure 2C

**Figure 3:**
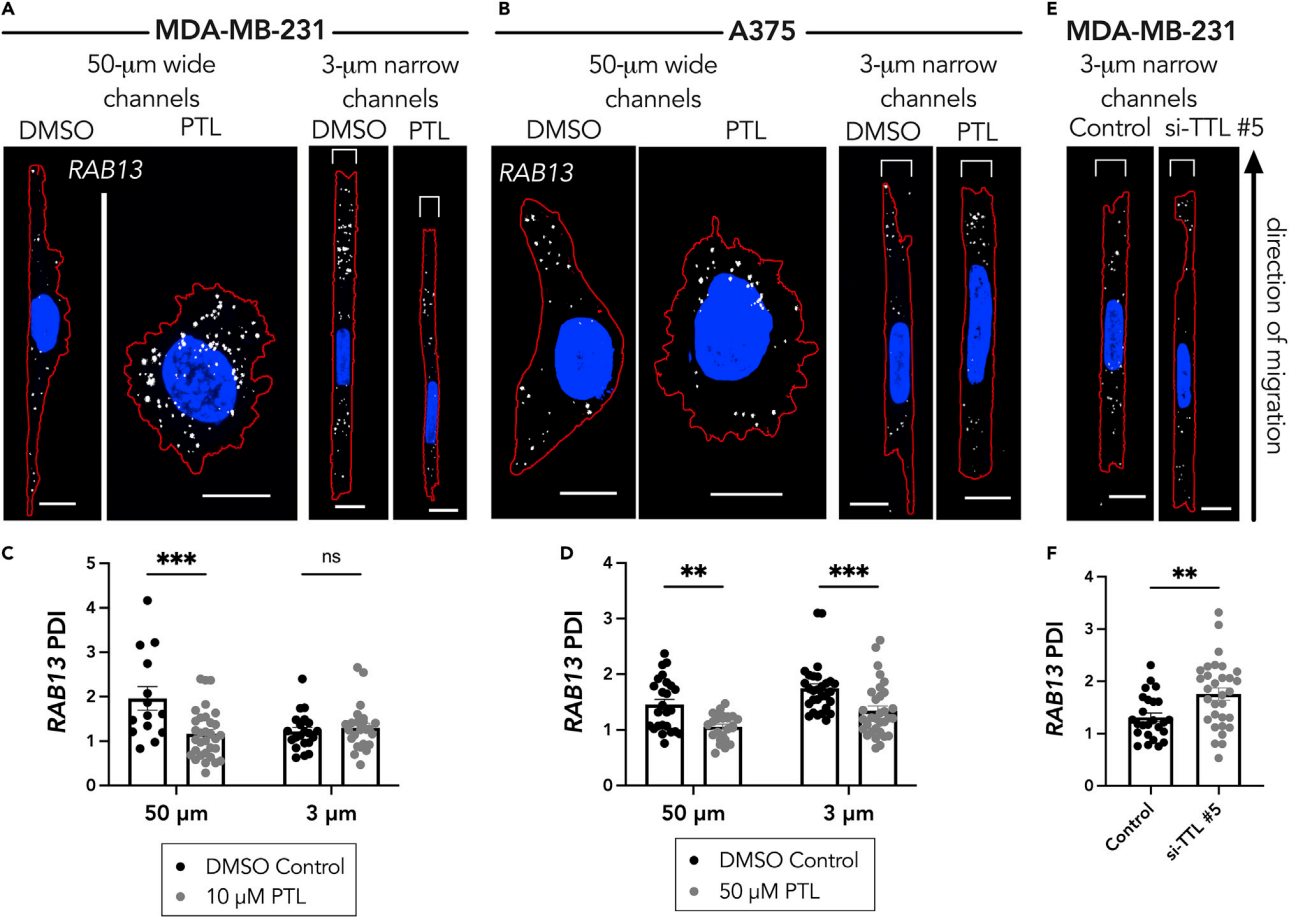
Glu tubulin levels drive *RAB13* RNA localization in cells in confinement (A and B) Representative maximum intensity z-projected *RAB13* FISH images of (A) MDA-MB-231 cells and (B) A375 cells in 50-μm wide and 3-μm narrow microchannels after 3-h treatment with DMSO (vehicle control) or Parthenolide (PTL; 10 μM in (A) and 50 μM in (B)). RNA signal is shown in white, nucleus in blue, and cell outline in red. Brackets above cells in 3 μm groups denote the microchannel width. Scale bars: 10 μm for wide channel images and 5 μm for narrow channel images. (C and D) PDI calculations of *RAB13* RNA distribution of (C) MDA-MB-231 cells and (D) A375 cells. (E) Representative *RAB13* FISH images of MDA-MB-231 cells treated with control siRNA or TTL siRNA. (F) PDI calculations of *RAB13* RNA distributions of MDA-MB-231 cells treated with control or TTL siRNA. Individual dots on graphs in (C, D, and F) represent individual cells pooled from three independent experiments (n = 14–32); bars display mean ± SE. p Values, ∗∗<0.01, ∗∗∗<0.001 by two-way ANOVA with Šídák’s test (C and D) or by unpaired t test assuming equal standard deviations (F).

To directly test whether Glu-tubulin is involved in peripheral RNA localization in cells in confinement, we took advantage of the fact that we can modulate the levels of Glu-tubulin by manipulating the enzymes involved in the α-tubulin tyrosination/detyrosination cycle (Figure 2A). Tubulin carboxypeptidase, the enzyme that cleaves the tyrosine at the end of the α-tubulin monomer, can be pharmacologically inhibited with parthenolide (PTL), leading to a reduction in detyrosinated tubulin (Figures S2A and S2B). Consistent with prior reports for cells on 2D substrates (Wang et al., 2017), in unconfined 50-μm wide microchannels, treatment with PTL reduced the peripheral localization of the *RAB13* RNA in both MDA-MB-231 and A375 cells (Figures 3A–3D). In the 3-μm narrow microchannels PTL reduced the peripheral *RAB13* localization in A375 cells but did not affect its distribution in MDA-MB-231 cells (Figures 3C and 3D). Therefore, the remaining Glu-tubulin network in confined A375 cells is functionally important to support peripheral RNA localization, whereas the fragmented network seen in MDA-MB-231 cells does not have a determining impact on the already less localized *RAB13* RNA.

To test whether we could rescue Glu-tubulin formation and consequently RNA localization in confined MDA-MB-231 cells, we employed siRNA-mediated knockdown of the tubulin tyrosine ligase (TTL) protein (Figures 2A and S2C). TTL restores the tyrosine at the end of the Glu-tubulin monomer; therefore, its knockdown can increase the levels of Glu-tubulin. Indeed, treatment of MDA-MB-231 cells with the TTL siRNA led to an increase in the visible detyrosinated tubulin network in 3-μm narrow microchannels (Figures S2D and S2E). This also led to a significant increase in the *RAB13* PDI in the confined MDA-MB-231 cells (Figures 3E and 3F). Together these data show that detyrosinated microtubules support the peripheral localization of the *RAB13* RNA in confined A375 cells, whereas the absence of detyrosinated microtubules underlies the reduced *RAB13* localization observed in confined MDA-MB-231 cells.

### Myosin II activity alone does not promote peripheral RNA localization in cells in confinement

Previous work from our laboratory showed that mechanical properties and actomyosin contractility can drive formation of the detyrosinated tubulin network, leading to peripheral RNA localization in cells (Wang et al., 2017). We speculated that differences in these parameters could account for the differences observed in Glu-tubulin levels and RNA localization between cell types. First, we investigated YAP distribution since higher nuclear YAP has been shown to correlate with a more mechanoresponsive cell (Dupont et al., 2011; Elosegui-Artola et al., 2017; Rianna et al., 2020). In the 50-μm wide microchannels, there was a high degree of nuclear YAP in both cell lines (Figures S3A and S3B), such that the cytoplasm to nuclear signal ratio was close to 1. In the 3-μm narrow microchannels, the MDA-MB-231 cells excluded YAP from the nucleus, leading to a high cytoplasm to nuclear signal ratio. A375 cells in 3-μm narrow microchannels also had increased cytoplasmic YAP in comparison with cells in 50-μm wide microchannels, but they maintained a higher relative level of nuclear YAP in comparison with MDA-MB-231 cells. These results are consistent with the idea that A375 cells may maintain a higher degree of mechanoresponsiveness in confinement compared with MDA-MB-231 cells.

**Figure 4:**
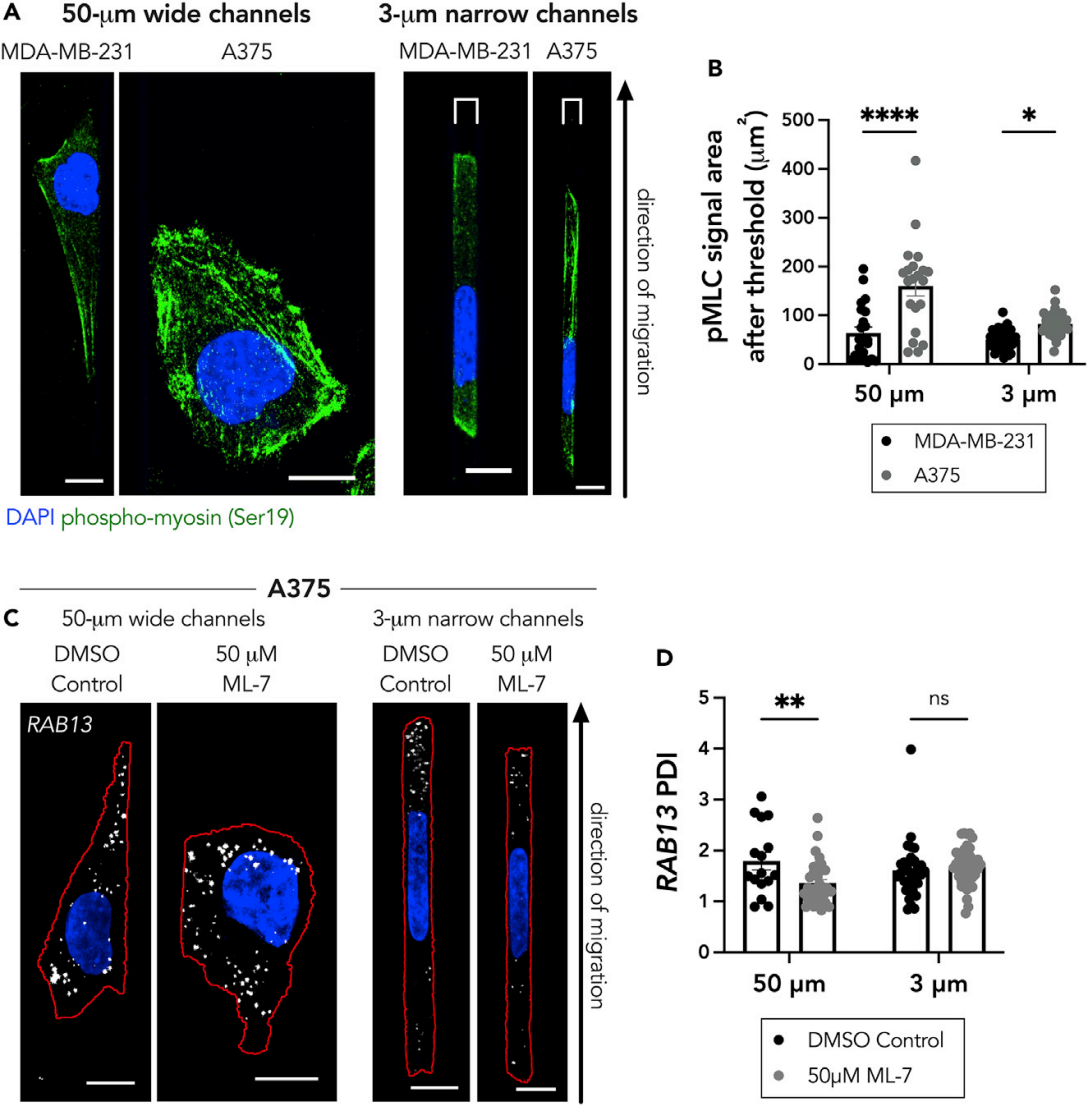
Loss of myosin activity is not sufficient to disrupt peripheral *RAB13* RNA localization in cells in confinement (A) Representative maximum intensity z-projected images of phospho-myosin light chain (Ser19) (pMLC) staining in MDA-MB-231 and A375 cells in 50-μm wide and 3-μm narrow microchannels. Brackets above cells in 3 μm groups denote the microchannel width. (B) Quantification of pMLC signal (after thresholding out diffuse background signal) of MDA-MB-231 and A375 cells in 50-μm wide and 3-μm narrow microchannels (n = 22–37). (C) Representative maximum intensity z-projected *RAB13* FISH images of A375 cells treated with DMSO (vehicle control) or 50 μM ML-7 in 50-μm wide and 3-μm narrow microchannels. RNA signal is shown in white, nucleus in blue, and cell outline in red. (D) Quantification of *RAB13* PDI of DMSO control or 50 μM ML-7 treated cells. Individual dots on graphs represent individual cells pooled from three independent experiments (n = 16–43); bars display mean ± SE. p Values, ∗<0.05, ∗∗<0.01, ∗∗∗<0.001 by two-way ANOVA with Šídák’s test. Scale bar: 10 μm.

To further test this, we explored the presentation of the actin and myosin networks within cells in confinement. We did not detect discernable differences in the actin network between either cell line in confinement (Figures S4A and S4B). In both cell lines in confinement, the actin network appeared largely cortical, which is a hallmark of ameboid migration that utilizes myosin II contractility. Staining with an antibody against active phosphorylated myosin (Ser19) revealed that both unconfined MDA-MB-231 and A375 cells displayed detectable active myosin, which concentrated in fibrillar structures, likely reflecting actin filaments (Figure 4A). In the 3-μm narrow microchannels, the A375 cells maintained detectable active myosin. Meanwhile, the MDA-MB-231 cells exhibited mostly background staining in the bulk cytoplasm and rather accumulated active myosin at the extreme peripheral edges of the cells. Quantification of the pMLC signal revealed significantly more active myosin in the A375 cells compared with the MDA-MB-231 cells in both the 50-μm- and the 3-μm-wide microchannels (Figure 4B). Interestingly, the levels of active myosin mirrored those of Glu-tubulin (compare Figures 4B and 2D), highlighting a potential connection between them.

To explore this possibility, we sought to disrupt myosin activity through treatment with the myosin light-chain kinase inhibitor, ML-7. We chose to use ML-7 in lieu of blebbistatin as it specifically targets myosin light-chain kinase, which phosphorylates the myosin light-chain residue that we imaged above. As expected, treatment with ML-7 led to a decrease in phosphorylated myosin signal (Figures S4C and S4D). Intriguing, when we examined the *RAB13* RNA patterns in these cells, we saw that, although myosin activity was required for *RAB13* RNA localization in unconfined microchannels, its loss was not sufficient to affect *RAB13* RNA localization in cells in confinement (Figure 4C). We conclude that, in confined A375 cells, a factor different from, or in addition to, myosin activity is required for peripheral RNA localization.

### The Piezo1 channel and myosin activity function redundantly to promote peripheral RNA localization in cells in confinement through the detyrosinated tubulin network

**Figure 5:**
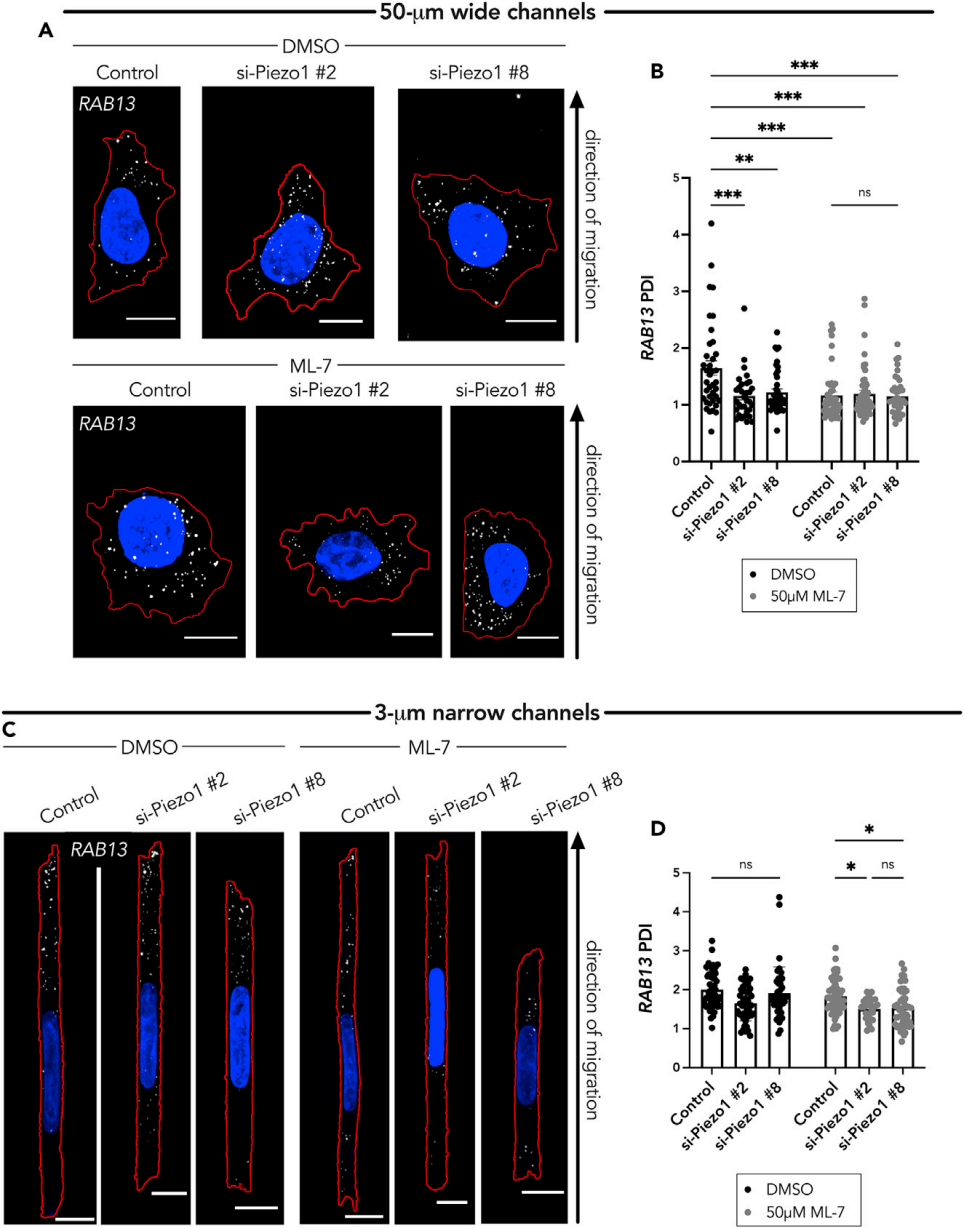
The Piezo1 channel and myosin activity function redundantly to regulate peripheral *RAB13* RNA localization in cells in confinement (A) Representative maximum intensity z-projected FISH images of *RAB13* RNA (in white dots, nucleus in blue, cell outline in red) in A375 cells in 50-μm wide microchannels treated with DMSO (vehicle control) or 50 μM ML-7 along with the indicated siRNAs. (B) Quantification of *RAB13* PDI of A375 cells in 50-μm wide microchannels treated with DMSO or ML-7 and Piezo1 siRNAs (n = 38–47). (C) Representative maximum intensity z-projected FISH images of *RAB13* RNA (in white dots, nucleus in blue, cell outline in red) in A375 cells in 3-μm narrow microchannels. Cells were treated with DMSO (vehicle control) or 50 μM ML-7, along with the indicated siRNAs. (D) Quantification of *RAB13* PDI of cells in 3-μm narrow microchannels (n = 28–55). Individual dots on graphs represent individual cells pooled from three independent experiments; bars display mean ± SE. Scale bars: 10 μm (A), 5 μm (C). p Values, ∗<0.05, ∗∗<0.01, ∗∗∗<0.001, ∗∗∗∗<0.0001 by two-way ANOVA with Šídák’s test.

It has been suggested that, in confinement, A375 cells maintain mechanoactivity through two independent factors, myosin II and the Piezo1 Ca^2+^ mechanosensitive ion channel, which initiates a downstream signaling cascade that controls myosin-mediated contractility (Hung et al., 2016). Only dual inhibition of these factors resulted in a change in cell stiffness on 1D micropatterned substrates (Hung et al., 2016). We speculated that the additional factor involved in peripheral RNA localization postulated above is the Piezo1 channel. Hence, we investigated the role of Piezo1 in RNA localization. Knockdown of Piezo1 with two different siRNAs (Figure S5A) reduced the peripheral *RAB13* RNA localization in cells within the 50-μm wide microchannels (Figures 5A and 5B). Combination of Piezo1 knockdown with 50 μM ML-7 treatment, in the 50-μm wide microchannels, did not lead to any additive effect. The RAB13 RNA remained perinuclear (evidenced by a significantly decreased PDI of approximately 1) compared with the control group.

In the 3-μm narrow microchannels, as seen above upon myosin inhibition, treatment with Piezo1 siRNA alone did not substantially alter the RAB13 PDI (Figures 5C and 5D). However, co-treatment with Piezo1 siRNA and 50 μM ML-7 significantly decreased the RAB13 RNA PDI (Figures 5C and 5D). Taken together, these results confirm that, in a confined environment, the Piezo1 Ca^2+^ mechanosensitive ion channel and myosin activity act redundantly to maintain peripheral RNA localization.

**Figure 6:**
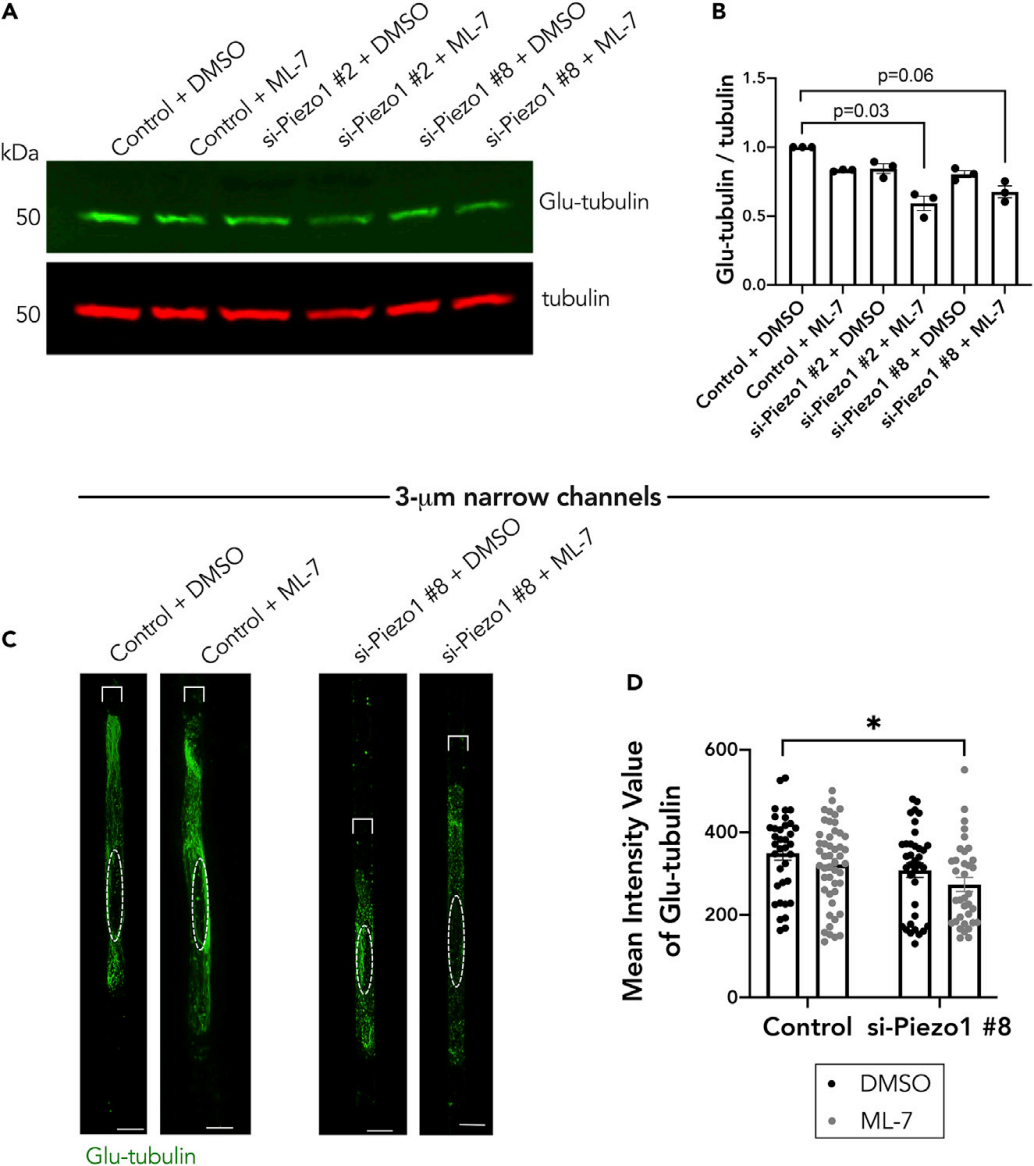
The Piezo1 channel and myosin activity function redundantly to regulate the detyrosinated tubulin network in cells in confinement (A) Representative western blot showing effect of Piezo1 knockdown and ML-7 co-treatment (DMSO is used as the vehicle control) on Glu-tubulin protein levels for cells on a 2D surface. (B) Quantification of corresponding Glu-tubulin to tubulin protein levels from (A); individual dots represent one independent experiment. (C) Representative immunofluorescence images of Glu-tubulin in A375 cells in 3-μm wide microchannels after treatment with Piezo1 siRNA and/or ML-7. Images are snapshots of 3D view renderings. Dashed white circle represents position of nucleus. (D) Quantification of mean intensity of Glu-tubulin signal per cell (n = 35–48). Dots represent individual cells pooled from three independent experiments. Bars reflect mean ± SE; scale bars: 5 μm. p values, ∗<0.05, by two-way ANOVA with Šídák’s test.

To determine whether this effect is mediated through the detyrosinated tubulin network, we assessed Glu-tubulin levels under single or dual inhibition of these factors. Indeed, only upon dual treatment with Piezo1 siRNA and ML-7 was there maximal decrease in Glu-tubulin levels as assessed by western blot (Figures 6A and 6B) or by immunostaining of A375 cells within the 3-μm narrow microchannels (Figures 6C and 6D). Only dual treatment with Piezo1 siRNA and ML-7 led to a significant decrease in the detyrosinated tubulin intensity (Figure 6D). The data suggest that there is a threshold of Glu-tubulin below which it is not sufficient to support *RAB13* RNA localization; however, we cannot exclude that there may also be a change in the organization/distribution of Glu-microtubules that also contributes to this effect. Altogether, the above data support a model whereby in confined A375 cells both Piezo1 mechanoactivity and myosin activity can promote the formation of a detyrosinated tubulin network and consequently lead to peripheral *RAB13* RNA localization.

### Peripheral RNA localization contributes to confined migration

Peripheral localization of the *RAB13* RNA is important for cell migration in 2D and 3D settings (Chrisafis et al., 2020; Moissoglu et al., 2020; Wang et al., 2017). Targeting of the *RAB13* RNA to the periphery can be specifically inhibited through the use of antisense morpholino oligonucleotides targeted against a functionally important GA-rich region within the 3′ UTR of *RAB13*. Such oligos have been shown to specifically mislocalize the *RAB13* RNA in MDA-MB-231 cells from peripheral regions of cells, without altering total *RAB13* RNA or protein levels (Moissoglu et al., 2020). We first confirmed that these tools produce the same results in A375 cells. Indeed, morpholinos targeted against the GA-rich region in the 3′ UTR of *RAB13* RNA prevented the peripheral localization of *RAB13*, as evidenced by a decrease in *RAB13* PDI, but did not alter the PDI of a perinuclear RNA, *RHOA* (Figures S6A–S6C). Significantly, and consistent with prior studies, RAB13 morpholinos also reduced cell migration speed and persistence of A375 cells on 2D unconstrained surfaces. These results support the conclusion that peripheral RAB13 is also important for migration of this cell type (Figures S6D and S6E). RAB13 morpholinos also prevented the peripheral localization of *RAB13* RNA in confinement (Figure S6F).

**Figure 7:**
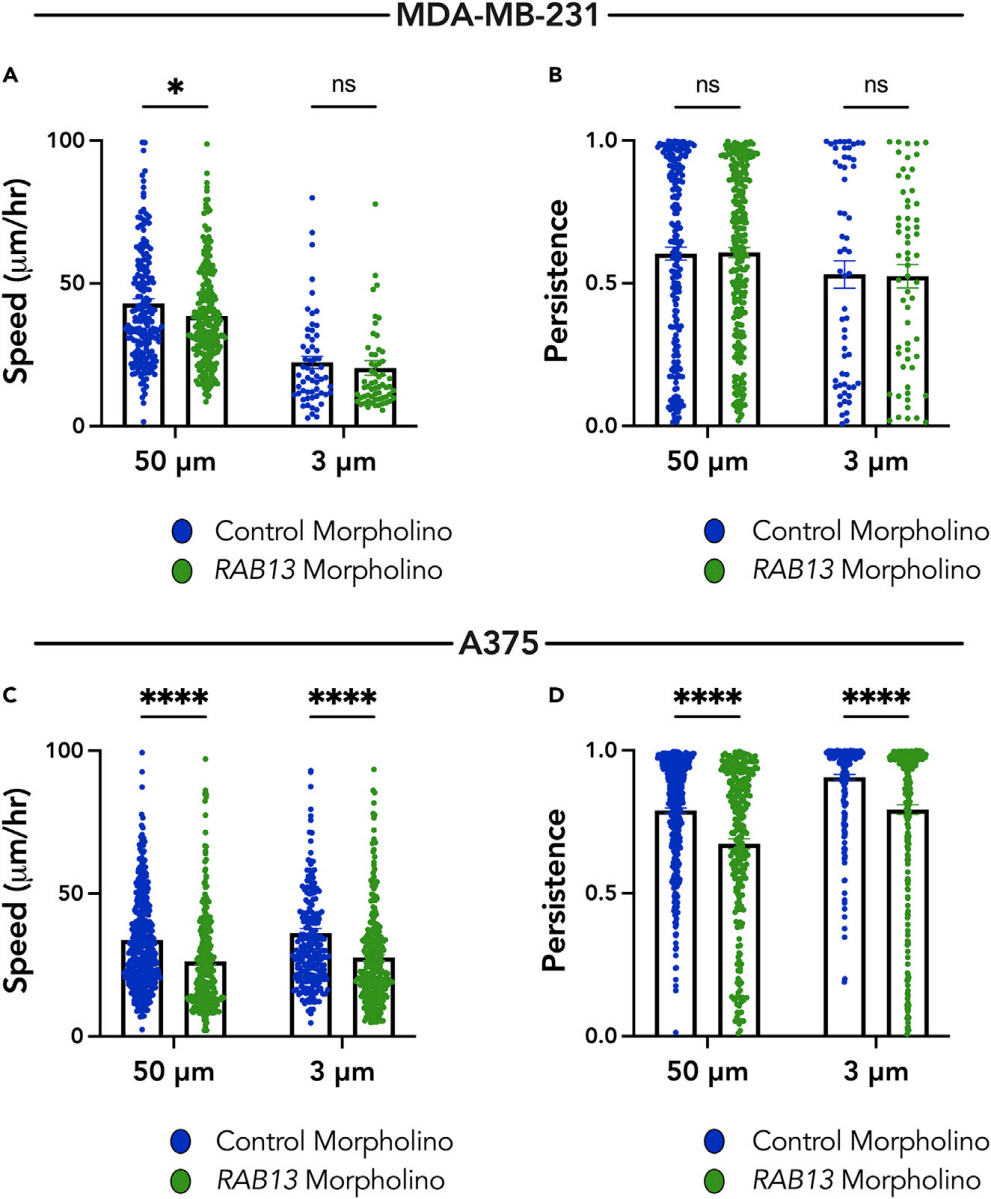
Peripheral RNA localization functionally contributes to A375 cell migration in wide and narrow channels (A and B) Cell speed (A) and persistence (B) of MDA-MB-231 cells in 50-μm wide and 3-μm narrow microchannels were quantified after treatment with control morpholino oligos or oligos directed against localization sequences in the 3′ UTR of *RAB13* RNA. (C and D) Cell speed (C) and persistence (D) of A375 cells in 50-μm wide and 3-μm narrow microchannels were quantified after treatment with control morpholino oligos or oligos directed against the 3′ UTR of *RAB13* RNA. Dots represent individual cells pooled from three independent experiments (n > 60). Bars represent mean ± SE. p values, ∗<0.05, ∗∗∗∗<0.0001 by two-way ANOVA with Šídák’s test.

To assess the role of *RAB13* RNA localization in confined migration, we seeded cells treated with antisense morpholino oligos against *RAB13* into the 50-μm wide microchannels or into the 3-μm narrow microchannels and quantified their migratory parameters. In the MDA-MB-231 cells, there was a significant decrease in cell speed in the 50-μm wide microchannels but no significant change in the 3-μm narrow microchannels when compared with treatment with control morpholinos (Figure 7A). There was no change in cell persistence in either case (Figure 7B). By contrast, there was a significant decrease in A375 cell speed and cell persistence, in both the 50-μm wide and 3-μm narrow microchannels, upon treatment with *RAB13* morpholinos (Figures 7C and 7D). Together, these data support a model where, in confinement, cells that maintain peripheral *RAB13* RNA localization (A375 cells) require this peripheral RNA accumulation for efficient migration. In contrast, cells that cannot support efficient peripheral RNA localization in confinement (MDA-MB-231 cells), due to a reduced Glu-tubulin network, likely rely on a different migratory mechanism that does not involve peripheral *RAB13* RNA.

## Discussion

**Figure 8:**
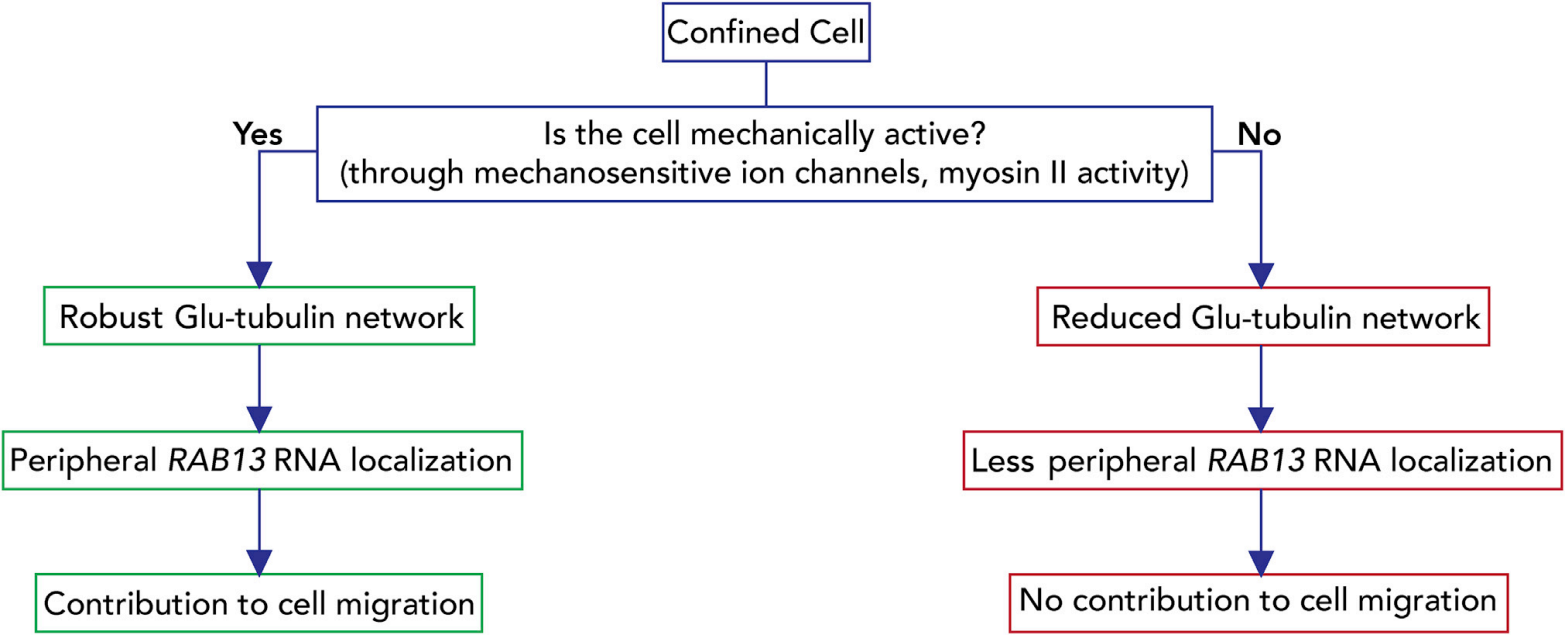
Proposed model Flow chart depicting how the mechanical properties of cells determine RNA localization in confined cells. Peripheral RNA targeting can promote migration through narrow spaces. We note that our data suggest that formation of a robust detyrosinated tubulin network is necessary to promote peripheral *RAB13* RNA localization. It is possible that mechanical activity might act through additional ways to affect cytoplasmic RNA distributions.

In summary, we have shown here that RNA localization in confinement is controlled by the mechanoactivity of cells. Mechanically active cells, through the Piezo1 channel or myosin activity, establish a detyrosinated tubulin network and utilize it to peripherally enrich the *RAB13* RNA. This peripheral RNA accumulation functionally contributes to cell movement through a confined space. Less mechanically active cells do not establish a pronounced detyrosinated tubulin network, do not manifest the same set of peripherally enriched RNAs, and do not require them for migration in confinement (Figure 8).

Cells in confinement have been reported to be less mechanically active than their unconfined counterparts. U2OS osteosarcoma cells soften as they move into confinement, with lower Young’s moduli and denser actomyosin stress fibers at the cortical edges of the cell (Rianna et al., 2020). Both NIH-3T3 fibroblasts and U2OS osteosarcoma cells in confinement exert lower traction forces compared with cells in unconfined environments (Raman et al., 2013). YAP localization can also be a marker of cell mechanoactivity, where greater nuclear YAP signal can correlate with a more mechanically active cell, whereas greater cytoplasmic signal can correlate with a “softer” cell (Dupont et al., 2011; Rianna et al., 2020). U2OS cells in confinement exclude YAP from the nucleus compared with nuclear accumulation of YAP in unconfined environments (Rianna et al., 2020). Our results suggest that residual cell mechanoactivity can vary in confinement depending on the cell type. Although both cell types tested here appear to be less mechanically active compared with unconfined settings, A375 cells maintain a higher degree of mechanoactivity compared with MDA-MB-231 cells. This difference is also reflected in different levels of active myosin, microtubule dynamics, and consequently RNA localization patterns. A375 cells are also able to move faster and more persistently in confined microchannels than MDA-MB-231 cells (Figure 7), suggesting a potential benefit of higher mechanoactivity in confinement. However, the underlying cause, purpose, and/or function of this difference warrants further investigation.

Entry of cells into a confined environment is accompanied by numerous changes in adhesion, cytoskeleton organization, and membrane tension, all of which alter the mechanical and signaling state of the cell and ultimately influence the adopted mode of migration. Localized increases in membrane tension can drive the opening of stretch-activated ion channels leading to an intracellular ion influx that can direct downstream signaling cascades (Sachs, 2010). A375 cells upregulate the Piezo1 stretch-activated ion channel, which allows entry of Ca^2+^ into the cytoplasm in response to changes in membrane tension (Hung et al., 2016). In confined cells, Piezo1-directed Ca^2+^ influx results in activation of phosphodiesterase 1 (PDE1), which suppresses protein kinase A (PKA) through hydrolysis of cAMP (Hung et al., 2016). We speculate that this signaling cascade could affect APC-dependent RNA localization by leading to activation of RhoA, as RhoA and PKA are connected through a negative feedback loop (Tkachenko et al., 2011). RhoA can, in turn, activate mDia1, a formin protein, which can impact both actin and microtubule dynamics (Bartolini and Gundersen, 2010). Confined A375 cells additionally maintain a residual level of active myosin, which can further promote RhoA activation (Hoffman et al., 2011; Lessey et al., 2012). Hence, it is possible that the combined effects of Piezo1 depletion and ML7 on *RAB13* RNA localization are due to Piezo1 depletion causing RhoA inhibition and therefore reduced ROCK signaling. In this case, Piezo1 could simply be acting synergistically to inhibit myosin II contractility.

In contrast to A375 cells, MDA-MB-231 cells do not upregulate Piezo1, but rather other mechanosensitive ion channels, such as transient receptor potential channels (TRPs) and Na^+^/H^+^ exchangers (Canales et al., 2019; Stroka et al., 2014). MDA-MB-231 cells in confinement can utilize the TRPM7 channel and cortical actomyosin to mechanically probe and enter environments of varying degrees of hydrostatic pressure, but their inhibition does not affect the cells' ability to migrate once in confinement (Zhao et al., 2019). Similarly, the MDA-MB-231 clone that has been purified from successive brain metastatic lesions (MDA-MB-231 BR) overexpresses the Piezo2 channel (Pardo-Pastor et al., 2018). In MDA-MB-231 BR cells, loss of Piezo2 does not impact confined migration, but rather the ability of the cells to enter confinement (Pardo-Pastor et al., 2018). These examples are in contrast to A375 cells, where knockdown of Piezo1 significantly reduces both confined migration and entry into confined microchannels (Hung et al., 2016). Together, it seems that mechanosensitive ion channels and actomyosin play cell type-dependent roles in confinement sensing and migration. It will also be interesting to determine whether differences in the degree of nuclear deformation and subsequent modulation of cell contractility through cytosolic phospholipase A_2_ might additionally underlie some of the cell type-specific differences described here (Lomakin et al., 2020; Venturini et al., 2020).

We show here that changes in mechanical activity and cytoskeletal dynamics underlie different RNA localization patterns in confined cells. Peripheral *RAB13* RNA localization is maintained in unconfined and confined A375 cells and is required for their efficient migration in both environments (Figure 7; Moissoglu et al., 2020; Wang et al., 2017). This functional contribution likely stems from localized RNA translation at the periphery. Indeed, translation at protrusions is important for protrusion stabilization (Mardakheh et al., 2015), and the *RAB13* RNA itself is translated in peripheral protrusions. Of interest, active *RAB13* translation is observed in extending regions, whereas translationally silent *RAB13* RNA is found in retracting areas (Moissoglu et al., 2019). The functional contribution of *RAB13* RNA silencing at retracting areas is still unclear; however, a mechanism has been described through which active *RAB13* translation, in lamelipodial regions, contributes to cell migration. Specifically, *RAB13* translation allows a co-translational interaction of the nascent RAB13 protein with its activator RABIF. This peripheral association is required to direct the RAB13 GTPase activity to support cell migration (Moissoglu et al., 2020). It would be interesting to explore whether an analogous mechanism occurs during confined migration and whether a similar or different set of RAB13 effectors are involved in confined versus unconfined migration. Our results suggest that, for MDA-MD-231 cells, which do not maintain peripheral *RAB13* translation in confinement, these potential RAB13 effectors are not relevant for the adopted migration mode.

An intriguing set of RNAs that become prominently peripheral in confinement, in both cell types tested here, encode ribosomal proteins (APC-independent RNAs). Their observed peripheral enrichment in both cases suggests that this might be a broader response upon entering confinement, regardless of the migration mode adopted by each cell type. This peripheral enrichment lacks directionality, occurring apparently randomly either at the front or back of cells, potentially indicating a lack of direct involvement in cell migration. Understanding the functional role of this phenomenon is hampered by our lack of knowledge of the underlying mechanisms of localization. Although peripheral r-protein RNA localization can be mediated through the RNA-binding protein LARP6 and sequences in the 5′ UTR (Dermit et al., 2020), there is not currently an available way to specifically interfere with this RNA localization pathway. Changes in the location of r-protein mRNAs in polarized cells have been correlated with changes in the efficiency of their translation (Dermit et al., 2020; Moor et al., 2017). Specifically, protrusion localization of r-protein RNAs increases ribosomal protein synthesis. Such an increased r-protein production can lead to enhanced ribosome biogenesis and consequently to an increase in overall protein synthesis (Dermit et al., 2020). Locally translated r-proteins could additionally participate in on-site ribosome remodeling, which could alter local ribosome functions (Shigeoka et al., 2019). The functional relevance of either scenario in confinement is unclear. It would be interesting to assess whether overall protein synthesis changes as cells enter confinement, what the contribution of r-protein RNA localization is, and the potential functional roles of this regulation.

In summary, our work provides evidence that specific mechanical properties of cells direct the type of migration mode adopted in confinement at least partly through directing cytoplasmic RNA distributions. This work adds to the emerging picture that subcellular RNA localization is regulated by both the internal and external mechanical environment of the cell and is functionally relevant for both 2D and 3D cell migration.

### Limitations of the study

Although the microchannel devices are useful for studying cell migration phenotypes in confined spaces, their pliability is not reflective of all physiological settings. Furthermore, they likely do not adequately model the potential contribution of matrix remodeling by cells as they navigate confined environments *in vivo*. Further technical advances could shed light on these aspects. The presented mechanistic understanding is based on studies of the APC-dependent RNA, *RAB13*. Given that RNAs belonging to the APC-dependent group generally require detyrosinated microtubules for localization (Moissoglu et al., 2019), we consider it likely that other RNAs of this group are regulated in a similar fashion, but future work would be needed to support that point. Finally, we focused on two cell lines that employ different migration mechanisms (myosin-based ameboid migration or actomyosin-independent migration) in confinement and that exhibit differing levels of mechanoactivity. An expansion of this work to include additional cell types could strengthen the connections between mechanoactivity in confinement, migration mode, and subcellular RNA distributions.

## STAR★Methods

### Key resources table

**Table:** 

REAGENT or RESOURCE	SOURCE	IDENTIFIER
**Antibodies**		

Rabbit polyclonal detyrosinated tubulin		
(1:250 IF, 1:1000 WB)	Abcam	Cat#: ab48389; RRID:AB_869990
Mouse monoclonal α-tubulin, clone DM1A		
(1:500 IF, 1:2500 WB)	Sigma	Cat#: T6199; RRID:AB_477583
Rabbit polyclonal phospho-myosin light chain 2 (Ser19)		
(1:100 IF, 1:1000 WB)	Cell Signaling Technology	Cat#: 3671; RRID:AB_330248
Rabbit polyclonal Tubulin tyrosine ligase (1:1000 WB)	Proteintech	Cat#: 13618-1-AP; RRID:AB_2256858
Rabbit monoclonal YAP (D8H1X) XP® (1:100 IF)	Cell Signaling Technology	Cat#: 14074; RRID:AB_2650491

**Chemicals, peptides, and recombinant proteins**		

Alexa Fluor™ 647 Phalloidin	ThermoFisher Scientific	Cat# A22287
ML-7	Sigma	Cat#: I2764
Parthenolide	Sigma	Cat#: P0667
Collagen type I	Sigma	Cat#: C3867
tridecafluoro-1,1,2,2,tetrahydrooctyl-1-trichlorosilane	Sigma	CAS: 78560-45-9

**Critical commercial assays**		

ViewRNA ISH Cell Assay Kit	ThermoFisher Scientific	Cat#: QVC0001

**Experimental models: Cell lines**		

MDA-MB-231	ATCC	HTB-26
A375	ATCC	CRL-1619

**Oligonucleotides**		

Morpholino: Control sequence: 5′-CCTCTTACCTCAGTTACAATTTATA-3′	GeneTools, LLC	PCO-StandardControl-300
Morpholino: RAB13 Sequence: 5′-TCTTTCACTTCCTCAATTCATTCCT-3′	GeneTools, LLC	Custom oligo
Morpholino: RAB13 Sequence: 5′-CCTTCCTTTCCTCCTCCCTCTCTTC-3′	GeneTools, LLC	Custom oligo
RAB13 FISH probe	Affymetrix	Cat#VA1-12225
KIF1C FISH probe	Affymetrix	Cat#VA1-3006735
RPL27A FISH probe	Affymetrix	Cat#VA1-16562-01
RPS20 FISH probe	Affymetrix	Cat#VA1-16561-01
siRNA targeting tubulin tyrosine ligase sequence: 5′-AGGAGTTCAATCAGTACCTAA-3′	Qiagen	Cat# SI03145856
siRNA targeting Piezo1 sequence: 5′-CAGCCTTGTATGCACCGTCAA-3′ sequence: 5′-CCGGCCCTGTGCATTGATTAT-3′	Qiagen	Cat# SI00383656 Cat# SI04759153

**Software and algorithms**		

RDI calculator	Stueland et al. (2019)	
Confined Migration code	This paper	
ImageJ	NIH	imagej.nih.gov/ij/download.html
Imaris 9.7	Oxford Instruments	imaris.oxinst.com
Prism 9	GraphPad	graphpad.com

**Other**		

Polydimethylsiloxane (Dow SYLGARD™ 184)	Ellsworth Adhesives	Cat#: 2065622
SU-8 3010	MicroChem Corporation	Cat#: Y311060
SU-8 3025	MicroChem Corporation	Cat#: Y311072

### Resource availability

#### Lead contact

Further information and requests for resources and reagents should be directed to and will be fulfilled by the lead contact, Kimberly M. Stroka (kstroka@umd.edu).

#### Materials availability

This study did not generate any new unique reagents.

### Experimental model and subject details

MDA-MB-231 cells (derived from female patient metastatic pleural effusion site of breast adenocarcinoma, ATCC) were cultured in Leibovitz’s L-15 medium (ThermoFisher Scientific) supplemented with 10% FBS (ThermoFisher Scientific) and 1% Penicillin/Streptomycin (1000 U/mL; ThermoFisher Scientific) in a 37°C incubator with 50% humidity and 0% CO2. A375 (derived from female patient with malignant melanoma, ATCC) cells were cultured in DMEM (ThermoFisher Scientific) supplemented with 10% FBS, 1% Penicillin/Streptomycin in a 37°C incubator with 50% humidity and 5% CO2.

### Method details

#### Microchannel fabrication & binding

Microfluidic devices were fabricated as previously described (Balzer et al., 2012; Tong et al., 2012). All fabrication was carried out in the University of Maryland Fabrication Laboratory. Briefly, two masks were designed in AutoCad, the first with the microchannel layer, and the second with the feed lines. A 10 μm layer of SU-8 3010 negative photoresist (MicroChem Corp) was spun onto a 4” silicon wafer. A MA-4 SUSS aligner was used to UV crosslink the SU-8 through the microchannel mask. Uncrosslinked photoresist was washed away with SU-8 developer (MicroChem Corp). Channel widths of 50 or 3 μm by 200 μm in length were confirmed using a profilometer. A second 50 μm layer of SU-8 3025 negative photoresist (MicroChem Corp) was spun onto the wafer and the mask containing the feed lines was aligned to the microchannel features and exposed to UV light. Any remaining uncrosslinked photoresist was again washed away with SU-8 developer. Wafers were silanized overnight with tridecafluoro-1,1,2,2,tetrahydrooctyl-1-trichlorosilane (Sigma) in a vacuum desiccator. Polydimethylsiloxane (Sylgard™ 184, Ellsworth Adhesives) was mixed at a 10:1 base to crosslinker ratio, poured over the silicon wafer, placed into a vacuum desiccator for 1 hour and then baked at 80°C for at least 2 hours. PDMS was removed from the wafer, cleaned with 100% ethanol and water, dried at 80°C for 5 minutes, and then plasma treated for 2.5 minutes. The PDMS devices were then bound to glass coverslips for 5 minutes and the PDMS coverslip unit was UV sterilized for 10 minutes.

#### Cell seeding into microchannels

20 μg/mL collagen type I (Sigma) was added to all wells of the microfluidic device and allowed to adsorb for 1 hour at 37°C or overnight at 4°C. All wells were washed 3 times with PBS for 5 minutes each. Cells were trypsinized for 5 minutes and centrifuged. 5x104 cells were resuspended in 25 μL of serum free media (basal media supplemented with 1% Pen/Step), added to the bottom inlet, and allowed to incubate for 5 minutes at 37°C. Any remaining cell media was removed, and serum-free media was added to the bottom three inlets, while FBS-containing media was added to the top inlet to act as the chemoattractant. For 2D control experiments, coverslips were coated with 20 μg/mL collagen type I for 1 hour and then washed three times with PBS for 5 minutes each. Cells were plated at 1x105 cells/mL and allowed to adhere.

#### Drug inhibitors

Cells were allowed to migrate through the microchannels for 3 hours and then media was replaced with serum-free or serum-containing media, both with drug. Cells were incubated with drug for another 3 hours, washed twice in PBS for 5 minutes each, and then fixed with 4% methanol-free paraformaldehyde for 20 minutes. Drug concentrations were as follows: Parthenolide (MDA-MB-231, 10 μM; A375, 50 μM), ML-7 (A375, 50 μM). DMSO was used as a vehicle control for all treatments.

#### Fluorescence *in situ* hybridization (FISH)

Cells were allowed to migrate through the microchannels for at least 3 hours but no longer than 10 hours. Cells were washed 2 times in PBS for 5 minutes each and fixed in 4% methanol-free paraformaldehyde for 20 minutes. FISH was performed with ViewRNA ISH Cell Assay kit (ThermoFisher Scientific) according to the manufacturer’s instructions, with adjusted incubation times. The following probe sets were used: human *RAB13* #VA1-12225; human *KIF1C* #VA1-3006735; human *RPL27*α #VA1-16562-01; human *RPS20* #VA1-16561-01. To detect *polyA* RNAs, LNA modified oligodT probe (30 nucleotides) labeled with ATTO-655 was added during hybridization at 100nM and all amplification steps at 50nM. We use *polyA* as a marker of all RNA sequences within the cell. The entirety of the cell was stained with a Cell mask stain to obtain the cell outline and nuclei were stained with DAPI.

#### Immunofluorescence (IF) & western blotting

Cells were allowed to migrate through the microchannels for at least 3 hours but no longer than 10 hours. Cells were washed 2 times in PBS for 5 minutes each and fixed in either 100% ice cold methanol (for Glu-tubulin and tubulin) or 4% methanol-free paraformaldehyde (all other proteins). Cells were again washed 3 times in PBS for 5 minutes each and blocked for one hour in PBS containing 5% goat serum and 0.3% Triton X-100. Cells were incubated with primary antibodies overnight, diluted in PBS containing 1% bovine serum albumin and 0.3% Triton X-100. The following day cells were washed 3 times in PBS for 5 minutes each and then incubated with Alexa-conjugated secondary antibodies (ThermoFisher Scientific) at 1:500 in PBS containing 1% bovine serum albumin and 0.3% Triton X-100. Cells were washed 3 times again in PBS for 5 minutes and then nuclei were stained with DAPI and imaged.

Western blots were blocked using Intercept TBS blocking buffer (Li-Cor) and incubated with primary antibodies at concentrations listed in the reference table, diluted in blocking buffer with 1% Tween-20 overnight at 4°C. Secondary antibodies conjugated with IRDye (LiCor) were used at 1:10,000 in blocking buffer with 1% Tween-20 for one hour at room temperature. Blots were imaged using a Li-Cor Odyssey imaging system.

#### siRNA knockdown

Cells were grown to 60-70% confluency and then transfected with 20 pmoles/mL of siRNAs using Lipofectamine RNAiMAX (ThermoFisher Scientific), following the manufacturer’s instructions. TTL siRNA and Piezo1 siRNA sequence #8 treated cells were analyzed 72 hours after transfection. Piezo1 siRNA sequence #2 cells were re-transfected with 20 pmoles/mL siRNA at 48 hours and analyzed at 96 hours after transfection. siRNAs used were: AllStars Negative control siRNA (Qiagen, cat# 1027281); si-TTL#5 (Qiagen, cat# SI03145856; target sequence: 5′-AGGAGTTCAATCAGTACCTAA-3′, si-Piezo1#2 (Qiagen, cat# SI00383656; target sequence: 5′-CAGCCTTGTATGCACCGTCAA-3′; si-Piezo1#8 (Qiagen, cat# SI04759153; target sequence: 5′-CCGGCCCTGTGCATTGATTAT-3′).

#### Antisense morpholino oligonucleotides

Cells were plated and allowed to attach overnight before antisense morpholino oligonucleotides (synthesized by GeneTools, LLC) were added to the cells at final concentration of 20 μM with Endoporter (GeneTools, LLC) delivery vehicle. Cells were analyzed 72 h post treatment.

#### Imaging

RNA FISH and IF samples were imaged using a Leica SP8 confocal microscope (with a HC PL APO 63X oil CS2 objective). Z-stacks were taken through the entire volume of the cell at an interval of 0.3 μm per step. Migration analysis was performed on an Olympus IX83 microscope (LUCPlan FLN 20X phase objective) with an environmental chamber to maintain appropriate growth conditions. Images were taken at 5 or 10-minute intervals for 12 hours.

#### Quantification and statistical analysis

RNA distribution indeces were calculated using RDI Calculator (Stueland et al., 2019). Briefly, the Peripheral Distribution Index (PDI) is derived by calculating the second moment of RNA pixel intensity positions relative to the centrod of the nucleus. To normalize for differences in cell morphology, the second moment of the RNA is divided by the second moment of a hypothetical uniform distribution, which is derived as the second moment of all pixels within the binary cell mask image. The Polarization Index (PI) is calculated by identifying the distance of the centroid of the RNA signal relative to the centroid of the cell, and normalized for cell size and shape. Distributions along the leading to lagging edge axis were performed using a custom MATLAB script. The script identifies the leading and lagging edges of a cell in a z-projected image; divides the intervening area into 10 sections of equal width and calculates the fraction of RNA intensity in each. Z-stack images of Glu-tubulin and tubulin IF were processed through the denoise.ai function of NIS Elements software, and fluorescent intensity within the 3D cell volume was quantified from 3D-view renderings using Imaris 9.7.0. All other IF images were quantified using ImageJ; phopsho-myosin images were thresholded to remove diffuse background signal before quantification. Cells for migration analysis were tracked using the Manual Tracking ImageJ plugin. Speed and persistence values were calculated using a custom MATLAB code by K.M.S. We measure persistence as the stepwise distance divided by the total distance the cell travelled, such that a persistence of 1 reflects a cell that moved in a straight line. All values are represented as mean ± standard error (SEM) and data represents three individual trials that have been pooled. Individual replicates generally show consistent trends, even though they might not independently reach statistical significance. Prism 9 (GraphPad) was used for all statistical analysis; the significance level was set at 0.05. For multiple comparisons, a two-way ANOVA with Šídák's test was performed. For single comparisons, an unpaired t-test assuming equal standard deviations was used.

## Data Availability

• All data reported in this paper will be shared by the lead contacts upon request. • The RDI Calculator has been described and is available (Stueland et al., 2019). The confined migration MATLAB code (by K.M.S.) supporting the current study has not been deposited in a public repository but is available from the corresponding author upon request. • Any additional information required to reanalyze the data reported in this paper is available from the lead contacts upon request.
