# Supplementary material for: RNA localization in confined cells depends on cellular mechanical activity and contributes to confined migration
Source: iScience. 2022 Feb 1;25(2):103845. doi: 10.1016/j.isci.2022.103845 (PMC8850802; doi:10.1016/j.isci.2022.103845)
Supplement: Document S1. Figures S1–S6 [file mmc1.pdf]

iScience, Volume *25*

Supplemental information

RNA localization in confined cells depends on cellular mechanical activity and contributes to confined migration

Rebecca A. Moriarty, Stavroula Mili, and Kimberly M. Stroka

Figure S1. RNA polarization metrics are altered in confinement in a cell type dependent manner, related to Figure 1.

(A and B) Polarization index of *RAB13*, *KIF1C*, *RPL27α*, *RPS20*, and *polyA* RNAs in (A) MDA-MB-231 and (B) A375 cells in 50 μm wide and 3 μm narrow microchannels. Individual dots represent individual cells pooled from at least three independent experiments. P values; *<0.05, **<0.01, ***<0.001, ****<0.0001, a two-way ANOVA with Šídák's test was performed.

(C and D) Quantification of the intensity of APC-independent RNAs (*RPL27α*, *RPS20)* compared to *polyA* RNA in (C) MDA-MB-231 and (D) A375 cells along the length of cells in 3 μm narrow microchannels. Bars (A and B) or data points (C and D) represent mean +/- standard error. (n>25).

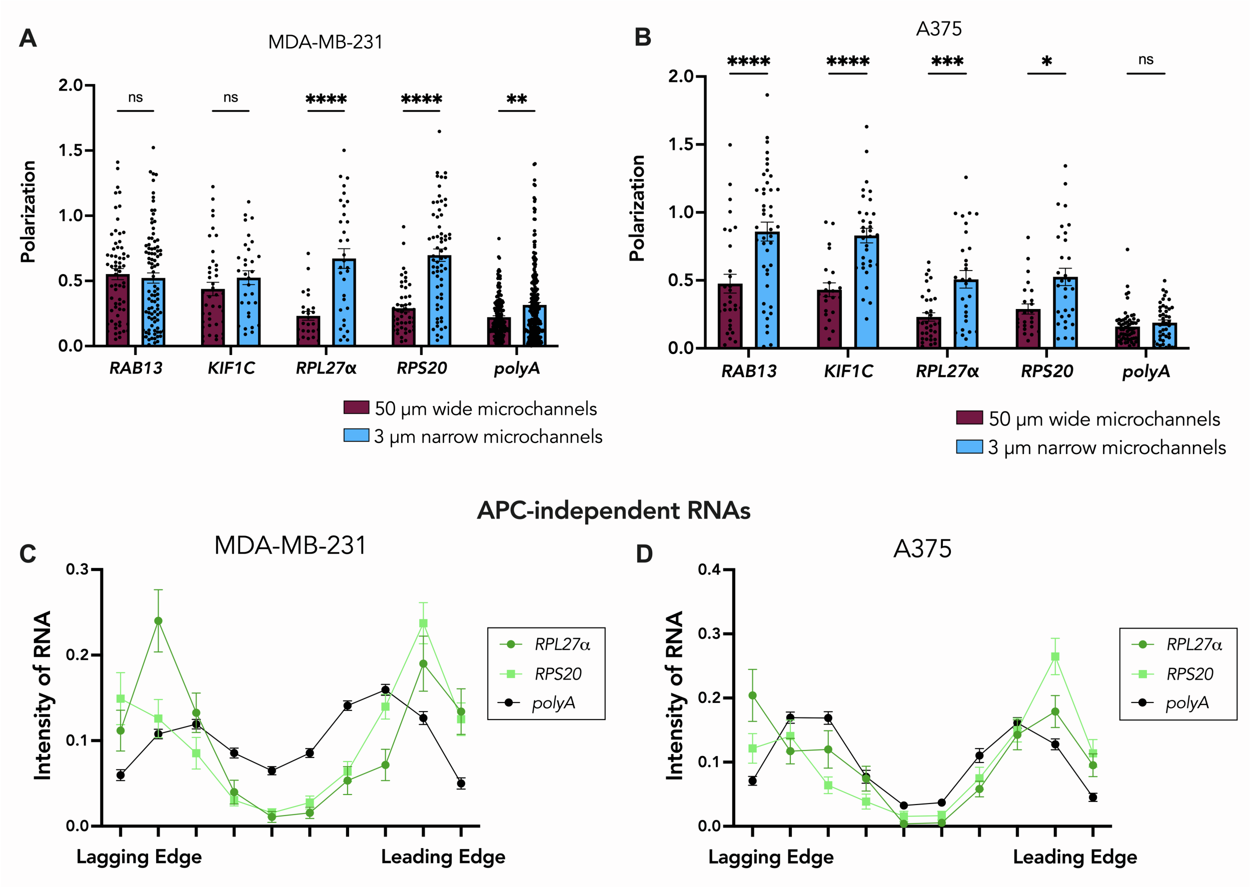

Figure S2. Modulation of Glu-tubulin levels, related to Figure 3.
(A and B) Western blots showing effect of DMSO (vehicle control) or Parthenolide (PTL) treatment on Glu-tubulin and tubulin protein levels for (A) MDA-MB-231 or (B) A375 cells. Numbers below tubulin bands represent quantification of Glu-tubulin to tubulin signal.
(C) Western blot showing tubulin tyrosine ligase (TTL) knockdown by siRNAs. Numbers below tubulin bands represent quantification of TTL to tubulin signal.
(D) Representative immunofluoresence images of MDA-MB-231 cells in 3 µm narrow microchannels treated with either Control or TTL siRNAs. Images are snapshots of 3D view renderings. White dashed lines represent position of the nucleus. Scale bar: 5 µm.
(E) Quantification of mean intensity of Glu-tubulin signal of Control or TTL siRNA treated MDA-MB-231 cells. Individual dots represent individual cells pooled from two independent experiments (n=22-29). P value, *<0.05, by unpaired t-test assuming equal standard deviations. Bars represent mean +/- standard error.

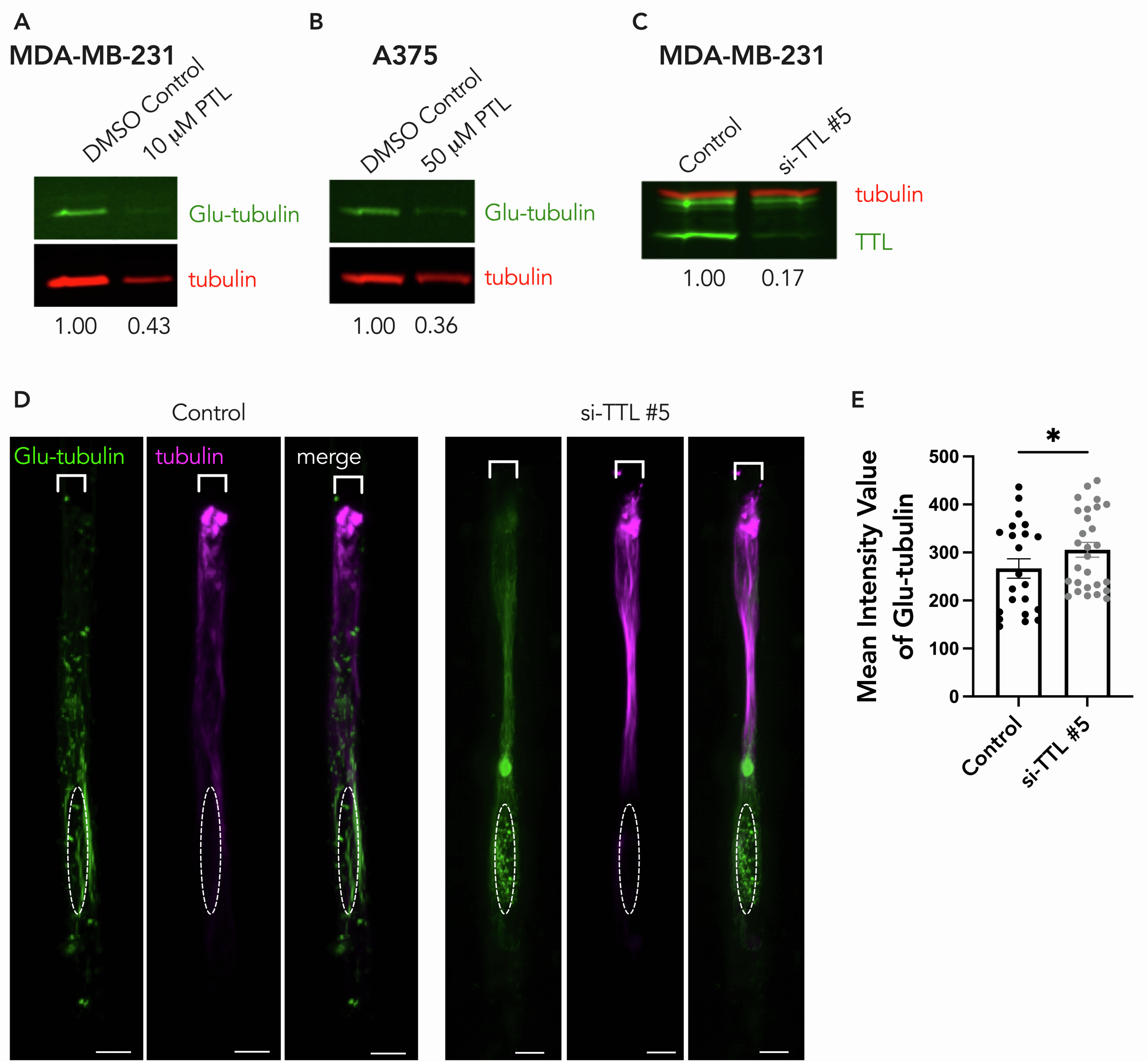

Figure S3. YAP localization is altered in cells in confinement, related to Figure 4.
(A) Representative immunofluoresence images of YAP staining in MDA-MB-231 and A375 cells in 50 μm wide and 3 μm narrow microchannels. Images are snapshots of 3D view renderings. Scale bar: 10 μm. Brackets above cells in 3 μm narrow microchannels show channel outline.
(B) Quantification of YAP cytoplasmic to nuclear signal of MDA-MB-231 and A375 cells in 50 μm wide and 3 μm narrow microchannels. Individual dots represent individual cells pooled from two independent experiments. (n=13-23). P value, ****<0.0001, by two-way ANOVA with Šídák's test. Bars represent mean +/- standard error.

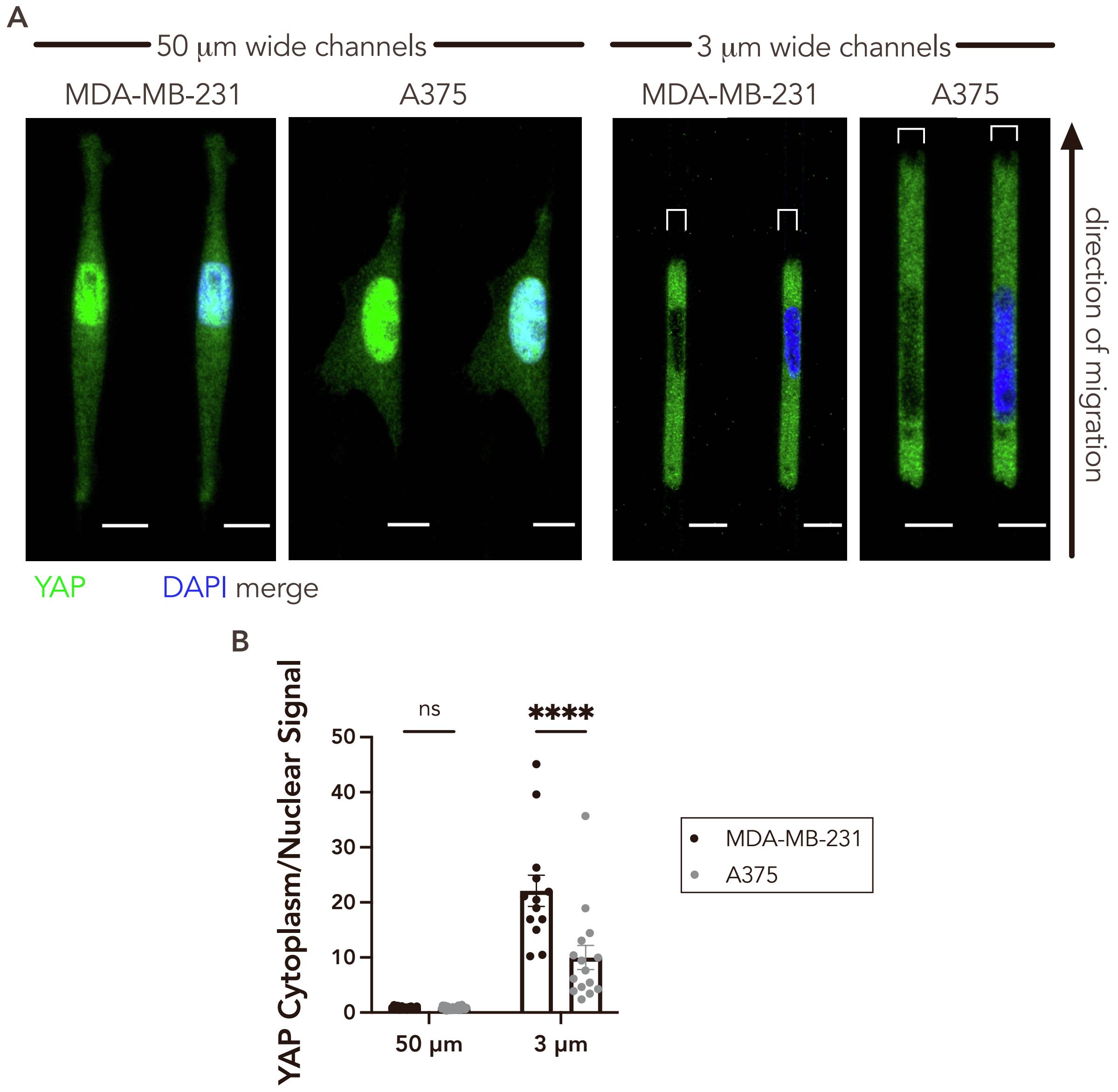

Figure S4. The actin network is largely cortical in cells in confinement and myosin light chain phosphorylation can be reduced by an inhibitor of the myosin light chain kinase, related to Figure 4.
(A and B) Representive immunofluorescent images of phalloidin staining in (A) MDA-MB-231 and (B) A375 cells in 50 μm wide and 3 μm narrow microchannels. Images are snapshots of 3D view renderings.
(C) Western blot of phosphorylated myosin light chain (pMLC) and GAPDH of A375 cells treated with DMSO (vehicle control) or 50 μM ML-7. Numbers below GAPDH band are quantification of pMLC/GAPDH signal.
(D) Representative immunofluorescent images of phospho-myosin light chain (Ser19) staining of A375 cells treated with DMSO (vehicle control) or 50 μM ML-7 in 50 μm wide or 3 μm narrow microchannels. Images are maximum intensity z-projections. Scale bar (A, B, and D): 10 μm.

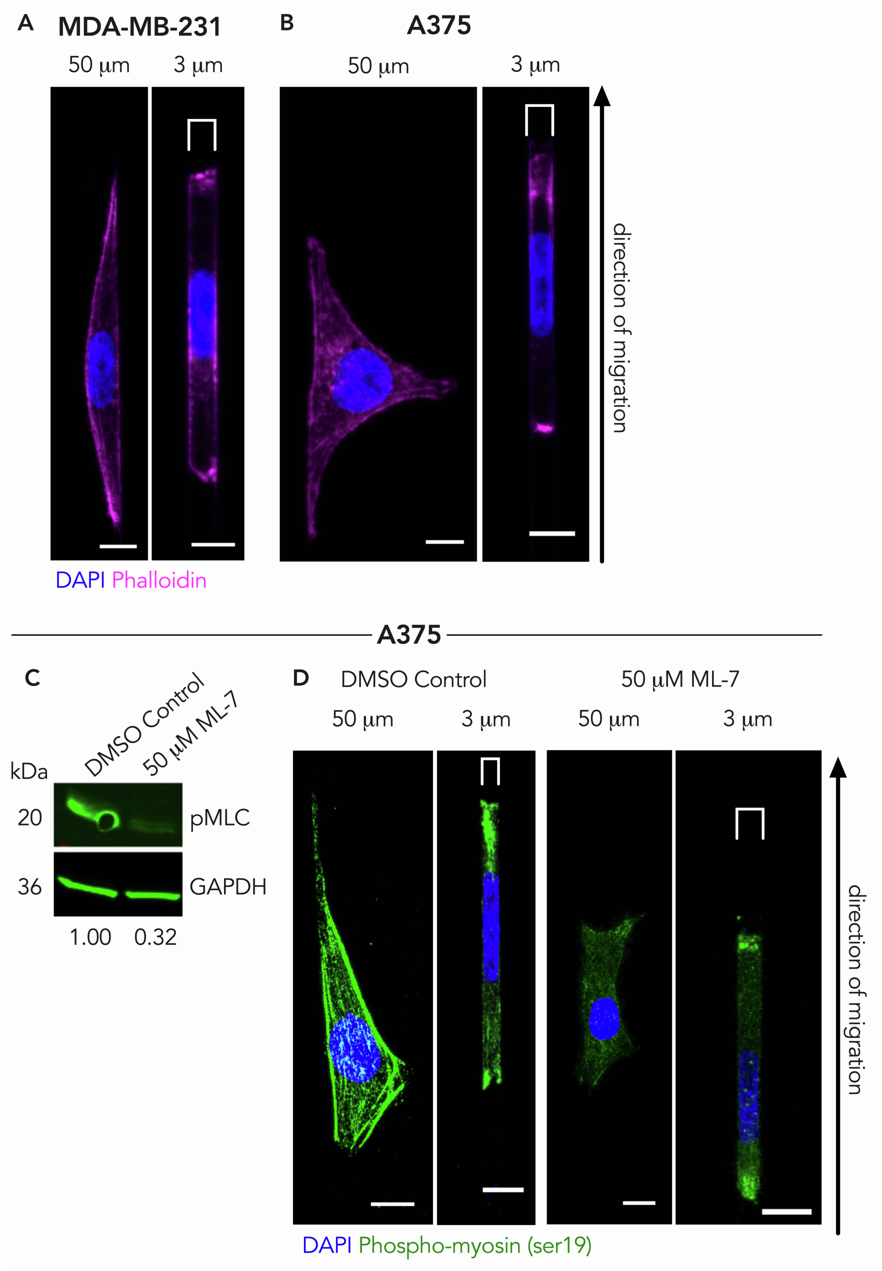

Figure S5. Piezo1 siRNA treatment reduces Piezo1 RNA levels, related to Figure 5.
(A) Quantification of RNA levels measured by droplet digitial PCR of Piezo1 compared to GusB housekeeping RNA of A375 cells treated with indicated siRNAs. Individual dots represent one experiment. (n=2). Bars represent mean +/- standard error.

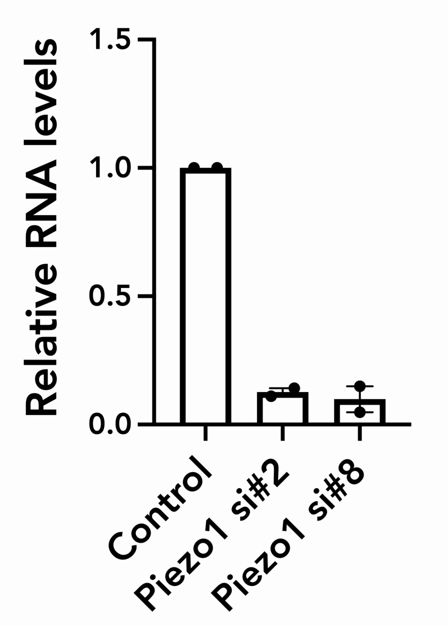

Figure S6. Antisense morpholino oligos targeted against localization sequences in the 3'UTR of *RAB13* can mislocalize the *RAB13* RNA and impact random 2D cell migration in A375 cells, related to Figure 7.

(A) PDI quantification of *RAB13* or *RHOA* RNAs in A375 cells treated with control morpholino oligos or *RAB13*-targeted oligos. Individual dots represent individual cells pooled from two independent experiments. (n=36-42).

(B and C) Representative maximum intensity z-projected *RAB13* FISH images of A375 cells treated with (B) control morpholino oligos or (C) oligos directed against localization sequences in the 3'UTR of *RAB13*. Cell outline is in red and nucleus in blue. Blue arrows in (B) depict peripheral localization of *RAB13* RNA, while yellow arrows in (C) show lack of *RAB13* RNA at cell protrusions.

(D and E) Effect of treatment of A375 cells with control morpholino oligos or oligos directed against localization sequences in the 3'UTR of *RAB13* on (D) cell speed and (E) persistence. Individual dots represent individual cells pooled from two independent experiments. (n=106-120).

(F) PDI quantification of *RAB13* RNA or *KIF1C* RNA, as a control, in A375 cells cells in 3 μm narrow microchannels treated with control morpholino oligos or *RAB13*-targeted oligos. Individual dots represent individual cells pooled from three independent experiments. (n=52-56).

Scale bar: 10 μm. P values, **<0.01, ***<0.001, ****<0.0001, by two way ANOVA with Šídák's test (in A, D, E, F). Bars represent mean +/- standard error.

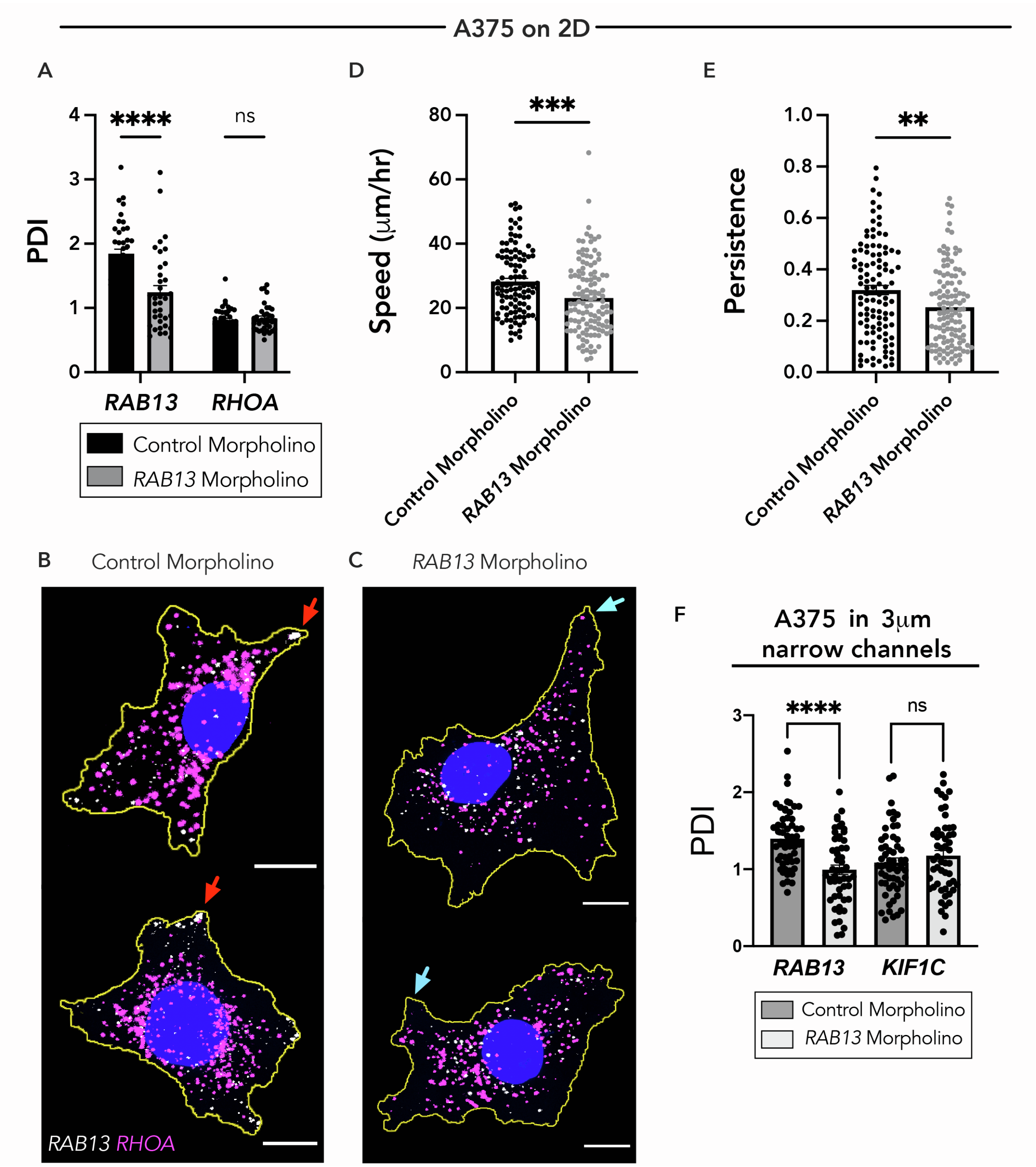

A375 on 2D
A
D
E
PDI
****
ns
RAB13
RHOA
Control Morpholino
RAB13 Morpholino
Speed (μm/hr)
***
Control Morpholino
RAB13 Morpholino
Persistence
**
Control Morpholino
RAB13 Morpholino
B
Control Morpholino
C
RAB13 Morpholino
RAB13 RHOA
F
A375 in 3μm narrow channels
PDI
****
ns
RAB13
KIF1C
Control Morpholino
RAB13 Morpholino
